# Quantitative in vivo phosphoproteomics reveals reversible signaling processes during nitrogen starvation and recovery in the biofuel model organism *Chlamydomonas reinhardtii*

**DOI:** 10.1186/s13068-017-0949-z

**Published:** 2017-11-28

**Authors:** Valentin Roustan, Shiva Bakhtiari, Pierre-Jean Roustan, Wolfram Weckwerth

**Affiliations:** 1 Department of Ecogenomics and Systems Biology, University of Vienna, Althanstrasse 14, 1090 Vienna, Austria; 2 Vienna Metabolomics Center (VIME), University of Vienna, Vienna, Austria

**Keywords:** *Chlamydomonas reinhardtii*, Nitrogen depletion, Nitrogen recovery, Phosphoproteomics, System-level analysis, Chloroplast, Protein translation regulation, TOR, AMPK, Plant systems biology

## Abstract

**Background:**

Nitrogen deprivation and replenishment induces massive changes at the physiological and molecular level in the green alga *Chlamydomonas reinhardtii*, including reversible starch and lipid accumulation. Stress signal perception and acclimation involves transient protein phosphorylation. This study aims to provide the first experimental phosphoprotein dataset for the adaptation of *C. reinhardtii* during nitrogen depletion and recovery growth phases and its impact on lipid accumulation.

**Results:**

To decipher the signaling pathways involved in this dynamic process, we applied a label-free in vivo shotgun phosphoproteomics analysis on nitrogen-depleted and recovered samples. 1227 phosphopeptides belonging to 732 phosphoproteins were identified and quantified. 470 phosphopeptides showed a significant change across the experimental set-up. Multivariate statistics revealed the reversible phosphorylation process and the time/condition-dependent dynamic rearrangement of the phosphoproteome. Protein–protein interaction analysis of differentially regulated phosphoproteins identified protein kinases and phosphatases, such as DYRKP and an AtGRIK1 orthologue, called CDPKK2, as central players in the coordination of translational, photosynthetic, proteomic and metabolomic activity. Phosphorylation of RPS6, ATG13, and NNK1 proteins points toward a specific regulation of the TOR pathway under nitrogen deprivation. Differential phosphorylation pattern of several eukaryotic initiation factor proteins (EIF) suggests a major control on protein translation and turnover.

**Conclusion:**

This work provides the first phosphoproteomics dataset obtained for *Chlamydomonas* responses to nitrogen availability, revealing multifactorial signaling pathways and their regulatory function for biofuel production. The reproducibility of the experimental set-up allows direct comparison with proteomics and metabolomics datasets and refines therefore the current model of *Chlamydomonas* acclimation to various nitrogen levels. Integration of physiological, proteomics, metabolomics, and phosphoproteomics data reveals three phases of acclimation to N availability: (i) a rapid response triggering starch accumulation as well as energy metabolism while chloroplast structure is conserved followed by (ii) chloroplast degradation combined with cell autophagy and lipid accumulation and finally (iii) chloroplast regeneration and cell growth activation after nitrogen replenishment. Plastid development seems to be further interconnected with primary metabolism and energy stress signaling in order to coordinate cellular mechanism to nitrogen availability stress.
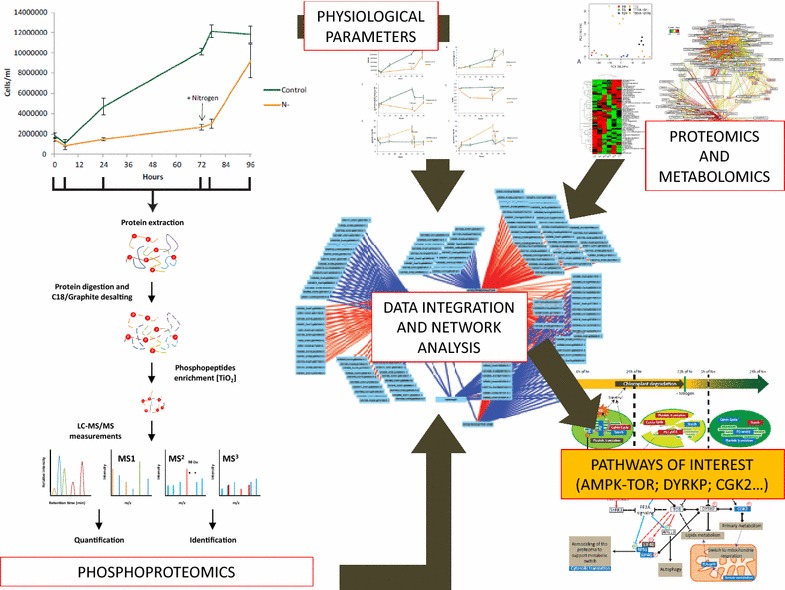

**Electronic supplementary material:**

The online version of this article (10.1186/s13068-017-0949-z) contains supplementary material, which is available to authorized users.

## Background

Microalgae are powerful CO_2_-neutral sources for biofuel/bioethanol/biomass production since they can convert solar energy, carbon dioxide, and wastewater into massive pools of carbon [1, 2] such as triacylglycerol (TAG) and starch [3, 4]. At the same time they do not compete with arable land for nutritional crops. This is especially important because nutritional crops are still used for biomass and biofuel production which is politically irresponsible with respect to decreasing plant productivity in global climate change and hunger crisis in poor regions of the world [5]. Accordingly, fundamental research on algal metabolism and physiology with respect to biomass and biofuel accumulation is a most pressing demand [5]. Among microalgae, *Chlamydomonas reinhardtii* (*Chlamydomonas*) has been extensively studied as a model organism [5–12]. The availability of a sequenced genome [13], various genetic tools [14, 15], a rapid growth rate, and tolerance to environmental changes (e.g., light intensity) has made these algae an attractive model for investigating a wide range of fundamental and industrial applications such as biofuel/bioethanol production [15]. Research on algae growth under nutrient limitation conditions has indicated that nitrogen (N) deprivation results in TAG and starch accumulation which are accompanied by significant physiological and structural reprogramming [16–18]. To understand the adaptive mechanisms of *Chlamydomonas* to N depletion, several studies have used a combination of metabolomics, proteomics, and transcriptomics tools along with physiological measurements to identify the pathways involved in lipid accumulation during N starvation [19–22]. Molecular processes during the recovery phase, however, have been neglected in these studies although starch and lipid accumulation is completely reversible by nitrogen replenishment [7]. Previously, we have addressed this question by setting up an N depletion and recovery experiment and applying a combined proteomics, metabolomics, and physiological analysis strategy to infer systemic correlation networks and provide a better understanding of cell adaptation and recovery [7]. Many processes involved in post-translational and degradation processes were identified indicating the intimate involvement of signaling by protein kinases [7]. Indeed, orchestration of the cell adaptation to environmental stimuli is known to be mediated by signaling processes, which regulate protein activity through post-translational-modification (PTM) [23]. Among the different forms of signal perception and transduction, reversible protein phosphorylation controlled by protein kinases and phosphatases is one of the most fundamental processes. Kinases and phosphatases are involved in intricate networks to adjust the transcriptome, proteome, and metabolome to environmental changes. Intriguingly, despite the importance of protein phosphorylation in rapid stress perception and signaling, the characterization of the phosphoproteome dynamics during N stress has never been studied for *Chlamydomonas*. Therefore, there is little knowledge about *Chlamydomonas* signaling pathways and potential regulatory processes, especially those implied in N stress adaptation. How important these signaling processes are has been demonstrated by Quantitative Trait Locus (QTL) analysis identifying a QTL acting as suppressor of carbon reserve accumulation during optimal growth [24]. A forward genetic screen identified a dual-specificity tyrosine-phosphorylation-regulated kinase-1, a homolog of the yeast kinase Yak1, as a regulator of TAG accumulation under nitrogen and sulfur stress [25]. Another plant-specific DYRK mutant (DYRKP) was identified as accumulating more oil than the wild-type under control and nitrogen deprivation conditions [26]. Concomitantly to these targets, the conserved TOR (Target of Rapamycin) and SnRK1 (Sucrose-non-fermenting-related kinase-1)/Snf1 (Sucrose-non-fermenting kinase-1)/AMPK (AMP-dependent-activated kinase) kinases, which control growth based on nutrient and energy availability (AMPK negative control, TOR positive control), are also involved in stress signaling [6, 7, 27–30]. Recently, we have defined and analyzed the AMPK orthologs in *Chlamydomonas* CKIN 1, 2, and 3 under cold stress acclimation [6]. In eukaryotic systems, an antagonistic crosstalk of AMPK–TOR signaling is proposed with AMPK upstream of TOR [29, 31]. TOR is a protein kinase which promotes protein translation and growth when enough nutrients are available [32]. In *Arabidopsis*, *At*TOR RNAi lines show a growth inhibition while starch and lipids accumulate [33]. Inhibition of TOR in *Chlamydomonas* triggered growth inhibition, while starch and TAG accumulate [34]. Interestingly, in *Chlamydomonas*, phenotypic and metabolomic observations between N stress and rapamycin treatment are similar [35, 36]. Further it was shown in *Chlamydomonas* that TOR inhibition like nitrogen deprivation induces autophagy through the accumulation of ATG8 protein [35, 37]. As autophagy is a dominant process in stressed *Chlamydomonas* cells, we have addressed this question as well by analyzing protein phosphorylation in nitrogen depletion and recovery experiments.

In the present study, we used an experimental set-up initially designed by Valledor et al. [7] and applied shotgun phosphoproteomics for the identification and quantification of the in vivo phosphoproteome [29, 38, 39]. Stability and robustness of molecular and physiological parameters between our measurements and those obtained by Valledor et al. indicate a remarkable reproducibility of the experimental set-up enabling a confident integration of phosphoproteome data with both proteomics and metabolomics data [7]. The obtained phosphoproteomics data were subjected to functional annotation, statistical analysis and integration with metabolomics, proteomics, and physiological data to provide a system-level analysis of stress perception and transduction. Significant changes in protein phosphorylation pattern were further investigated with the help of STRING/protein–protein interaction networks [40]. Results reveal a highly dynamic adaptation of the phosphoproteome during nitrogen depletion and inverse processes during the recovery phase. Sparse-Partial-Least-Square (SPLS) network analysis was used to integrate phosphoproteomics data with both physiological and proteome data [7]. These data mining processes revealed a multifactorial, sequential, and reversible reprogramming of primary metabolism, starch, and lipid accumulation, as well as chloroplast growth inhibition and degradation characterized by specific in vivo signaling pathways. Most specifically, these results highlight the central role of DYRKP and AMPK–TOR signaling pathways as key coordinators of the *Chlamydomonas* metabolism to N availability and as potential targets of interest to study regulatory processes as well as energy stress signaling in green algae in the context of starch and lipid accumulation.

## Results

### Characterization of *Chlamydomonas reinhardtii* growth and physiological parameter during nitrogen depletion and re-addition

**Fig. 1:**
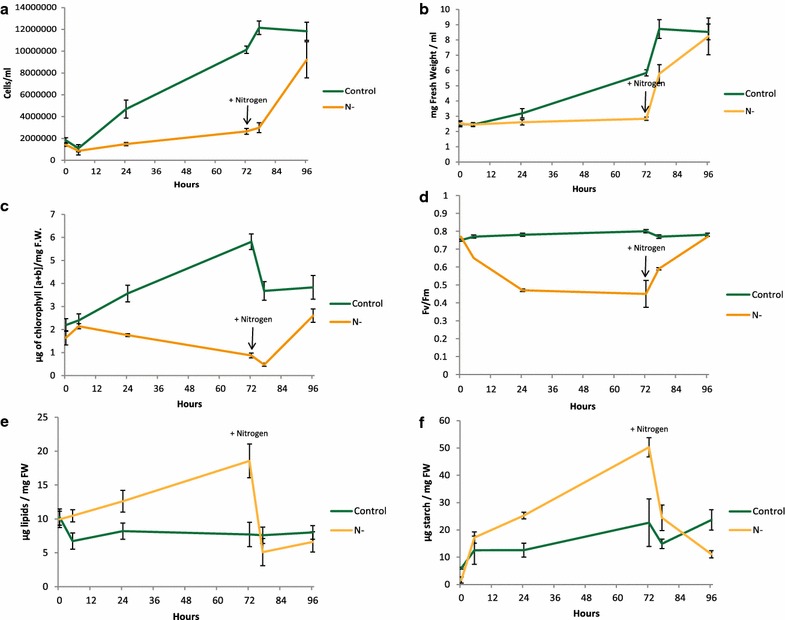
Phenotyping of *Chlamydomonas* under N depletion and recovery. **a** A time course (0, 5, 25, 72, 77, 96 h) was sampled and cell number was measured as well as variation in the fresh weight (**b**). Chlorophyll a plus b concentration (**c**) and maximum quantum efficiency of photosystem II (Fv/Fm) (**d**) were measured for each time point. Total lipids amount (**e**) and starch measurement (**f**) in *C. reinhardtii* under nitrogen stress (Up to 72 h) and nitrogen recovery (72–96 h) (*n* = 4)

To validate the reproducibility of the experimental set-up for nitrogen depletion and recovery proposed by Valledor et al. [7], multiple physiological parameters were measured in a time-course experiment. The experiment started when cells were transferred in a nitrogen-free medium (sampling time point 0 h) and covered a short- and long-term acclimation to low nitrogen availability (sampling time points 5, 24 and 72 h). For the recovery, NH_4_Cl (7 mM) was added after 72 h (sampling time points 77 and 96 h). Physiological measurements included growth rate, monitored as cell number (CN) and fresh weight (FW), photosynthetic efficiency (Fv/Fm), chlorophyll (Chl) content as well as starch and total lipid content (Fig. 1) (See “Methods”).

Nitrogen-depleted culture growth is significantly inhibited from 0 to 72 h when compared to the control culture growth rate (Fig. 1a, b). Chlorophyll content decreased from 1.7 µg Chl mg^−1^ FW to less than 0.9 µg mg^−1^ FW after 72 h of N deprivation (Fig. 1c). Chlorophyll fluorescence detection was used to measure the maximum quantum efficiency of photosystem II (PSII), referred to as Fv/Fm, during N deprivation (Fig. 1d). Fv/Fm decreases during the first 24 h (from 0.75 to 0.5), while in the control samples Fv/Fm stays stable (Fig. 1d). Fv/Fm results corroborated the results observed previously [7, 41, 42]. Figure 1e, f shows the total lipid content and starch concentration during nitrogen depletion and replenishment. In 72 h, nitrogen-depleted samples accumulated 1.68-fold more lipid and 10.6-fold more starch than the control condition. Lipid accumulation was found to take place mainly between 24 and 72 h while starch content showed an increase from 5 to 72 h of N depletion (Fig. 1e, f).

N replenishment quickly reverted these physiological adaptations. During the first 5 h of nitrogen replenishment, fresh weight presents a twofold increase while cell number is almost stable (Fig. 1a, b). Finally, after 24 h of nitrogen recovery, the nitrogen-depleted samples reached 75% of the control condition cell number. The decrease in chlorophyll content observed in the first 5 h after nitrogen recovery (Fig. 1c) might be explained by a higher growth rate compared to chlorophyll biosynthesis. Indeed the total amount of chlorophyll at 77 h is similar to the 72 h time point (Additional file 1: Figure S1a). After 24 h of N re-addition, the chlorophyll content of the recovered samples is 25% lower than in the control cells. Chlorophyll results are in line with Fv/Fm recovery in about 24 h. During the N recovery phase, lipids and starch degradation occurred quickly in the first 5 h by approximatively 3.6- and 2.1-fold change.

### Comprehensive analysis of the *Chlamydomonas reinhardtii* in vivo phosphoproteome during nitrogen depletion and recovery

**Fig. 2:**
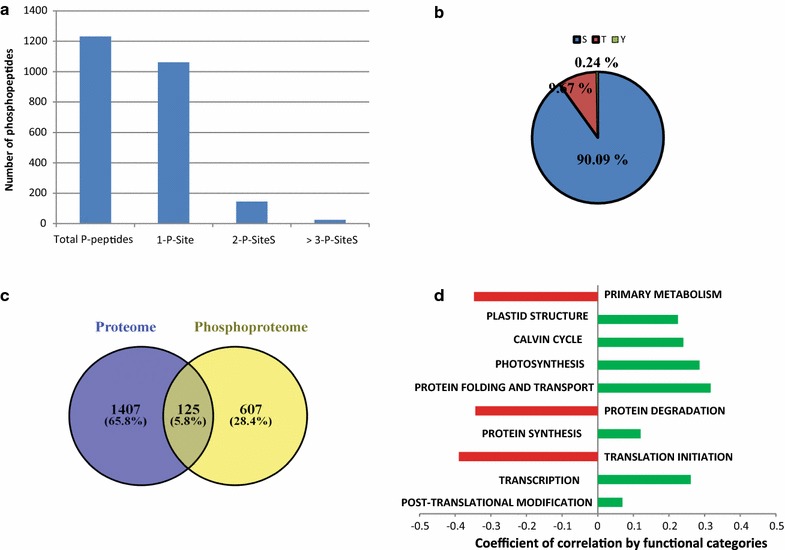
Summary of identified phosphosites, phosphopeptides, and phosphoproteins. **a** 89% of peptides with confidently assigned phosphosites were singly phosphorylated. **b** Majority of the 1227 confidently assigned phosphosites were pSer. **c** Overlap between the phosphoproteome and the proteome measured by [7], less than 10% of the proteome is covered by the phosphoproteome dataset representing 125 proteins. Coefficient of correlation between protein and corresponding phosphopeptides identified in **c** were calculated (Additional file 2: Table S3), in **d** the coefficient of correlation mean was calculated for each functional category excluding protein with unknown function

To allow a comprehensive in vivo phosphoproteome analysis of the *Chlamydomonas* nitrogen availability response and to unravel the interaction between the phosphoproteome dataset and phenotypic as well as proteomics data obtained by Valledor et al., a label-free in vivo shotgun phosphoproteomics approach based on LC–MS/MS technique was performed at the same time points as in Valledor et al. on nitrogen-depleted (0, 5, 24, and 72 h) and recovered (77 and 72 h) cultures. We applied a workflow which combines protein extraction, protein digestion, and a novel two-step peptide desalting procedure and subsequent phosphopeptide metal oxide affinity chromatography (MOAC) [43] enrichment to increase the coverage of identified and quantified phosphoproteins/-peptides [29]. From the generated dataset, only those of the phosphopeptides which were present in at least 50% of the samples (9 out of 18) were selected for relative quantification, corresponding to a data matrix of 1227 phosphopeptides mapped to 732 proteins (Additional file 2: Table S1). Functional annotation of the phosphorylated proteins was performed by matching the identified phosphoprotein with the *Chlamydomonas* MapMan file according to [7]. Among quantified phosphopeptides, 1037, 145, 25 of them had single, double, triple, or more phosphorylated peptides, respectively (Fig. 2a). The distribution of phosphorylated Ser, Thr, and Tyr residues were 1105, 119, and 3 respectively (Fig. 2b). A one-way ANOVA test identified 470 phosphopeptides that changed in abundance (threshold ≥ 1.5-fold) in at least one time point over the nitrogen depletion/recovery experiment (*P* < 0.05) (Additional file 2: Table S2).

Since it is not clear whether the detected site-specific phosphorylation changes originate from variation in kinase/phosphatase activities or due to fluctuations at the protein level, we compared the N stress phosphoproteome of *Chlamydomonas* with the proteomic results that were obtained previously for the same experimental design [7]. 125 proteins (corresponding to 276 phosphopeptides) showed an overlap (Fig. 2c, and Additional file 2: Table S3). From the phosphopeptides which showed significant changes, 125 belonged to a previously quantified protein [7]. In the following paragraphs, phosphoproteins that were found to overlap with the proteomics data have been marked with a star (e.g., Cre17.g720250*) (Additional file 2: Table S3 shows the phosphopeptides and their corresponding protein level measure by *Valledor* et al. [7]). Additionally, Pearson coefficients of correlation were calculated between the phosphopeptides and their corresponding peptide levels (Additional file 2: Table S3), and the average of the coefficient of correlation for each functional category was calculated and plotted in Fig. 2d. Three functional categories presented a negative pearson coefficient of correlation average: primary metabolism, protein degradation, and translation initiation. The coefficient of correlation represents the dynamics of in vivo phosphorylation compared to the concentration of the corresponding unphosphorylated peptides/proteins. Accordingly, a negative coefficient of correlation reflects active phosphorylation or de-phosphorylation of the proteins in response to the conditions.

### Multivariate statistics reveal system-level effects in the phosphoproteome during nitrogen depletion and recovery

**Fig. 3:**
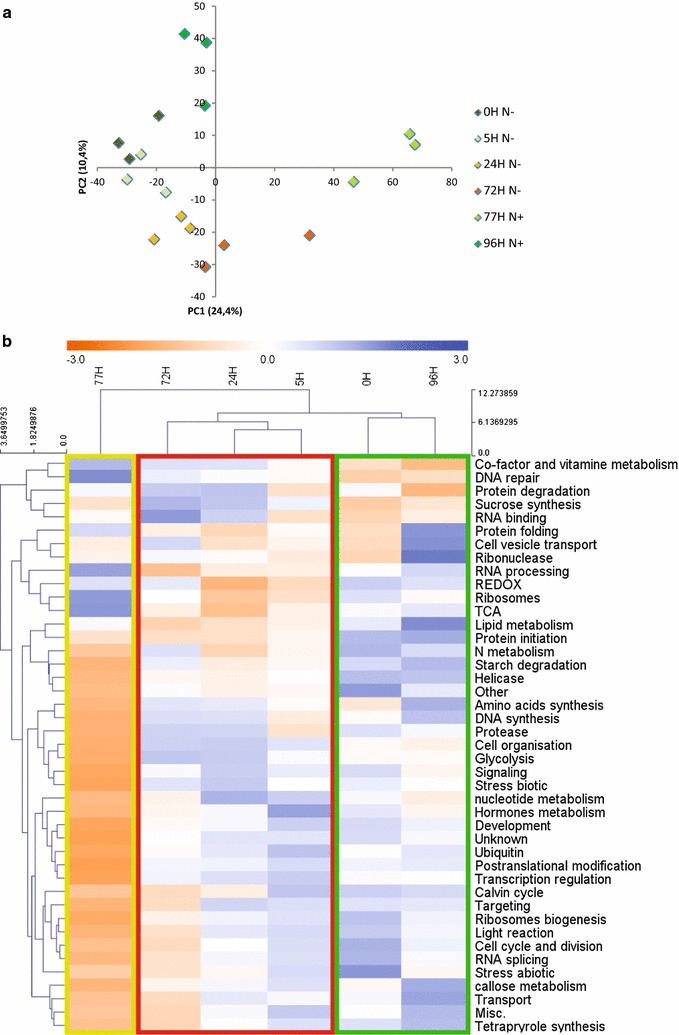
Classification of the time resolved phosphoproteome datasets according to multivariate methods. **a** Principal component analysis (PCA) of the phosphoproteomic dataset. Detailed information about loadings as well as relative levels of phosphopeptides is provided in Additional file 2: Table S4. **b** Hierarchical Clustering and heatmap analysis of functional protein categories according to MapMan. Three different clusters can be distinguished, showing the different degrees of response to N availability. Cluster 1 (green) comprising the recovered samples (0 and 96 h), Cluster 2 (red) comprising the N-depleted samples (5, 24, 72 h), and Cluster 3 (yellow-green) representing early recovered samples after N replenishment (77 h). Aggregation of the 96-h and 0-h time points is a result of cell recovery from N depletion by N replenishment. The bi-clustering uses average linkage of Euclidean distance between groups as the metric (Additional file 2: Table S5). (Control: 0 h; N depletion: 5, 24, and 72 h; N recovery: 77 and 96 h)

To study the phosphoproteome dynamics during nitrogen depletion and recovery, we applied univariate and multivariate approaches [5, 44]. Phosphopeptides detected in N-depleted and recovered samples (Additional file 2: Table S1) were subjected to principal component analysis (PCA) (Fig. 3a and Additional file 2: Table S4). PC1 separates sequentially the different time points of the experiment explaining the phosphoproteome adaptation over time. The strongest effects were observed on long-term adaptation to N depletion (72 h) and at the N recovery (77 and 96 h), while a remarkable recovery effect occured for the 96-h time point leading to a grouping with the 0 h. Exactly the same recovery effect was observed based on metabolome and proteome dynamics [7]. Here, phosphopeptides with the highest loading on PC1 are mostly involved in transcriptional processes, protein translation and degradation, cell organization, transport, and photosynthesis (Additional file 2: Table S4).

PC2, on the other hand, separates the samples based on nitrogen availability, explaining the difference in the phosphoproteome of nitrogen-depleted (5, 24, and 72 h) and recovered samples (0 and 96 h). Phosphopeptides with the highest loading on PC2 are involved in glycolysis, lipid, and amino acid metabolism, as well as protein translation (Additional file 2: Table S4). Hierarchical bi-clustering of functional categories of phosphopeptide changes (total phosphopeptide expression values were summed up for different functional categories and Z-standardized before mapping) (Fig. 3b and Additional file 2: Table S5) confirmed that the recovered cells cluster with 0 h cells suggesting a complete remodeling of the phosphoproteome to the cellular vegetative growth form as a result of nitrogen re-addition. This dynamic remodeling was also observed on the proteome and metabolome level by Valledor et al. [7] and it demonstrated the robustness of the experimental approach as well as the reversibility and plasticity of the molecular processes.

### Protein interaction and protein correlation network analysis revealed central protein kinases controlling signaling networks during nitrogen depletion and recovery

**Fig. 4:**
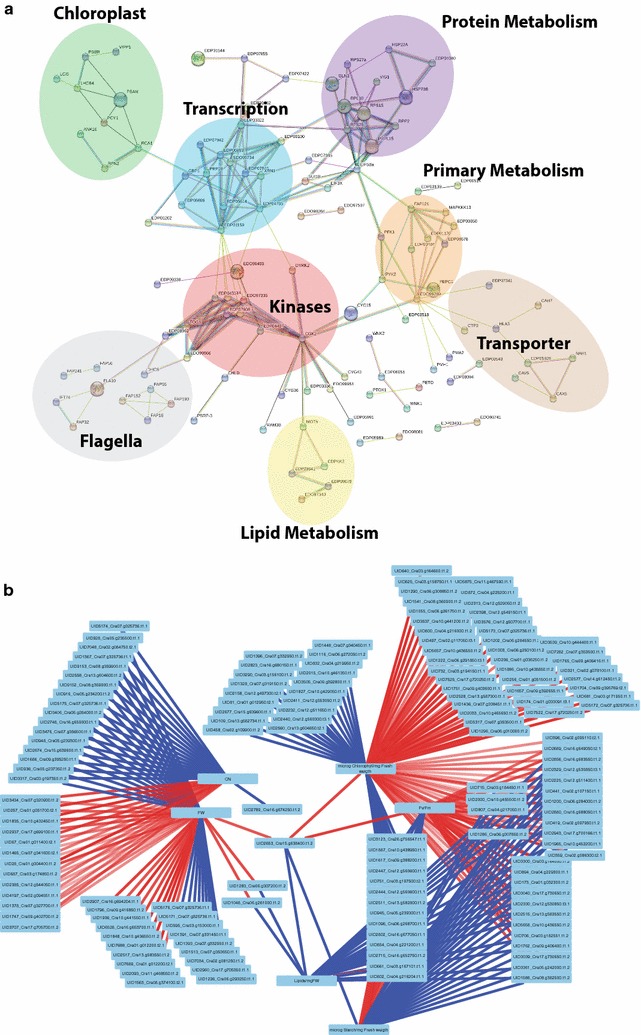
**a** Protein–protein interaction network of phosphoproteins kinases present in ANOVA datasets. Interaction networks were created using the STRING database for known and predicted protein–protein interactions (setting: medium confidence (0.4); http://string-db.org/). The list of protein sequences is provided in Additional file 2: Table S7; the highest percentage of identity was always chosen. **b** SPLS analysis of the predicted response phenotype based on phosphopeptides changes. SPLS analysis between significantly changed phosphorylation sites (Additional file 2: Table S2) and physiological parameters. Interaction network was visualized with Cytoscape. SPLS correlations are in the Additional file 2: Table S6. **b** Highlight only the correlation of an minimum absolute value of |0.6|

A STRING database search (see “Methods”) was used to predict protein–protein interaction network between the proteins which shows significant changes at their phosphosites level (Fig. 4a and Additional file 2: Table S2). The protein interaction network based on significantly changed phosphoproteins revealed major processes during adaptation to nitrogen depletion and recovery. Protein kinases are central in this network and connect several processes such as primary and lipid metabolism as well as chloroplast. Interestingly, protein kinases involved in cell cycle regulation are also connected to flagella-related proteins (Fig. 4a and Additional file 2: Table S2). To predict physiological changes from phosphoproteome dynamics, inference of correlation between these two datasets via SPLS analysis was conducted according to Valledor et al. [6, 7]. Phosphopeptides were used as a predictive variable while physiological measurements were used as response factors (Fig. 4b and Additional file 2: Table S6). Figure 4 b shows the correlation of a minimum absolute value of |0.6| between phosphopeptides and measured phenotypes. Only proteins for which a phosphopeptide was identified as one of the top 20 correlations (positive or negative) are further discussed (Additional file 2: Table S6). Three different groups are correlated to photosynthesis (Fv/Fm and chlorophyll content), cell growth (CN and FW), and carbohydrate storage (total starch and lipid content) (Fig. 4b). There is strong negative correlation between photosynthesis and carbohydrate storage groups which involve proteins such as EIF4-B (Cre16.g688050), RPL10 (Cre09.g388200). On the other hand, proteins negatively correlated to growth are CDPKK2 (Cre17.g705350) and ATG13 (Cre16.g659000) as well as cell vesicle-associated proteins such as Plastid lipid-Associated Protein (PAP—Cre07.g325736). Phosphopeptides belonging to lipid-related proteins such an acyltransferase (Cre07.g327700) and a phosphatidylcholine transfer protein (Cre03.g174850) showed positive correlation to growth.

### Consensus motif analysis of significantly changed phosphoproteins points to specific protein kinases involved in nitrogen stress adaptation

**Fig. 5:**
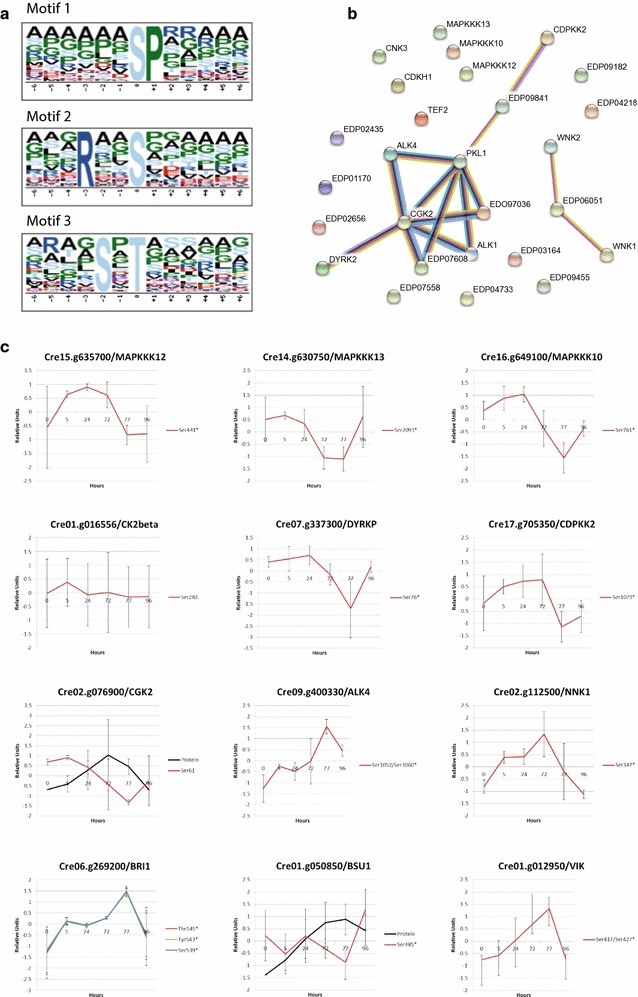
**a** Identification of regulated signaling pathways. **a** Motif-X analysis was done with significantly changed phosphopeptides in the one-way ANOVA. *P* value threshold was set to 0.0000001 and at least 10 occurrences were required. *Chlamydomonas* proteome was used as background proteome. **b** Protein–protein interaction network of phosphoproteins kinases present in both ANOVA and/or in common proteins datasets. Interaction networks were created using the STRING database for known and predicted protein–protein interactions [setting: high confidence (0.7); http://string-db.org/]. The list of protein sequences is provided in Additional file 2: Table S9. **c** Changes in phosphorylation of identified kinases and phosphatases. Phosphosites belonging to Kinases and Phosphatases present in the protein–protein interaction network in **b** and/or discussed in the text. In the protein–protein interaction network, DYRKP is DYRK2; NKK1 is EDP07608; and BSU1 is PKL1. Phosphopeptides are depicted in color while protein level is in black. Data represents Z-transformed normalized abundances of protein and phosphopeptides (*n* = 3 for phosphopeptides and *n* = 4 for protein level), Phosphosites followed by *are significant (Additional file 2: Table S2). Protein levels are based on data obtained previously [7]

To identify active signaling pathways during N depletion and recovery, a combination of three approaches was used. First, we searched for enriched protein phosphorylation motifs in significantly changed phosphopeptides by performing a motif-x analysis (http://motif-x.med.harvard.edu/motif-x.html) [45] (Fig. 5a). Second we used the STRING database to analyze a protein–protein interaction network between the proteins identified as kinases and phosphatases (Fig. 5b). Thirdly, a precise analysis of the phosphopeptide abundances was conducted across our experiment (Fig. 5c).

Among the three motifs significantly identified, the highest fold increase was 6.81 for motif 1 (SP) (Fig. 5a). Motif 1 is phosphorylated at a serine position directly followed by a proline. This phospho-motif is a low stringent motif recognized by Mitogen-Activated Protein Kinases (MAPKs) [46]. Plant mitogen-activated protein kinase (MAPK) cascades are composed of three types of reversibly phosphorylated kinases (MAP3K, MAP2K, and MAPK) leading to the phosphorylation of substrate proteins such as transcription factors. MAPK cascades play a central role in signaling; they transduce environmental signals into adaptive and programmed responses, including stress and developmental programs [47]. Enrichment of the MAPK motif highlights the central role of MAPKs to acclimate to nitrogen availability. Three mitogen-activated protein kinase kinase kinases (MAPKKK), out of 8–10 in *Chlamydomonas* [47] were detected as shown in Fig. 5a–c. Cre15.g635700, Cre14.g630750, and Cre16.g649100 presented a significant difference in their phosphorylation level. MAPKKK12 (Ser441) presented a phosphorylation increase during N depletion quickly reverted by nitrogen replenishment. Additionally, MAPKKK 13 (Ser2093) and MAPKKK10 (Ser761) presented together an approximately 1.5-fold de-phosphorylation level during N depletion (Fig. 5c). The second highest fold increase (3.08) was motif 2 (RXXS) with a serine at position 0 and a basic arginine at position − 3. This motif is known to be targeted by Ca2+/calmodulin-dependent protein kinase II (CAMKII) kinases which play a central role in biotic and abiotic stress response [48]. Finally, the third motif (SXXT) (2.15-fold change) presents a threonine residue at position 0 preceded by a serine residue at position − 2. This motif is targeted by Casein Kinases II (CKII) proteins involved in the cell cycle control as well as DNA repair [49, 50]. Only one non-significant phosphopeptide, belonging to a casein kinase II beta chain 2 (CKB2—Cre01.g016556), was found in our dataset (Fig. 5c).

Among the proteins present in the protein–protein interaction network, DYRKP and CDPKK2 are both related to energy metabolism and signaling. For instance, CDPKK2 (Cre17.g705350), a Calcium-Dependent Protein Kinase Kinase, was identified. CDPKK2 is related to energy sensing and signaling. Indeed, CDPKK2 is an orthologue of *Arabidopsis* GRIK1 (GEMINIVIRUS REP INTERACTING KINASE 1). AtGRIK1 specifically activates SnRK1 via phosphorylating the T-172 in its activation loop [51]. In our data, CDPKK2 presents a 1.9-fold phosphorylation increase up to 24 h at Ser1210 (Fig. 5b). Further, DYRKP (dual-specificity tyrosine-phosphorylation-regulated kinase) a protein involved in energy storage (Fig. 5a, b) [26], presented a 2.1-fold de-phosphorylation at Ser76 from 24 to 72 h (Fig. 5c). It was shown that DYRKP kinase inactivation strongly boosts accumulation of reserve compounds under photoautotrophic nitrogen deprivation [26].

Moreover, little is known about the implication of other protein kinases involved in *Chlamydomonas* cell cycle regulation. For instance, we identified CGK2 also called PKG (Cre02.g076900*—Ser61) (Fig. 5a, b). CGK2 is required for flagellar signaling through its activation in the flagellar by an unknown protein-tyrosine kinase [52]. Other protein kinases such as aurora kinases (AURs) and related proteins (ALKs) are described as flagellar activity and structure regulators in *Chlamydomonas* [53, 54]. Among them ALK4 (Cre09.g400330—Ser1052) and NNK1 (Nitrogen Network Kinase 1) (Cre02.g112500—Ser347) have the same phosphorylation pattern, respectively, they present a 2.4- and 2.1-fold increase of their phosphorylation level over the nitrogen stress followed by a decrease during N replenishment (Fig. 5c).

Finally, we identified several protein kinases and phosphatases related to the brassinosteroid (BR) signaling pathway. BR pathway is involved in chloroplast growth inhibition and degradation as well as in stress response [55]. A 2.9-fold increase in the phosphorylation of BRI1 (Brassinosteroid insensitive 1) protein was found at phosphosites Ser539, Thr545, Tyr543 over the N depletion time points (Fig. 5c). BRI1 is a receptor-like kinase located both in the plasma and nuclear membranes [56]. Additionally, no clear phosphorylation pattern for BR suppressor 1 (BSU1) protein was detected while the protein abundance of BSU1, measured by Valledor et al. [7], increases over the N depletion period (Cre01.g050850.t1.2—PLK1 in the network) (Fig. 5c). Associated to the BR pathway regulation, Cre01.g012950, a MAP3 K homolog to VH1-INTERACTING KINASE (VIK), was differentially phosphorylated at position Ser417 (Fig. 5c). Ser417 phosphosite presented a 2.2-fold increase in its phosphorylation pattern up to 77 h, before being down-regulated up to 96 h. VIK protein interacts physically with the receptor kinase BRL2/VH1 [57].

### Nitrogen availability regulates photosynthetic activity

From the measured phenotypic data, both the Fv/Fm and the chlorophyll content were found to decrease over N depletion indicating a clear inhibition of the photosynthetic activity (Fig. 1). More precisely Fv/Fm dramatically decreases in the first 5 h, suggesting that the PSII complex rapidly dissociates. Phosphoproteomics dataset suggests that phosphopeptides related to CP29 (Cre17.g720250*), a LHCII subunit, also show N-dependent regulation. CP29 contains various phosphorylation sites (Thr11, 17, 18, 27, 33) which are reported to be differentially regulated under different light conditions and particularly during highlight triggering PSII dissociation from the Antenna [58]. Here, all the indicated phosphopeptides are unchanged in the first 5 h while the protein content is decreasing. This result suggests a relative increase of the phosphorylation level for this specific phosphosite (Additional file 3: Figure S2B). In line with CP29 data, PsbR (Cre06.g261000*—Ser43), a protein related to the Oxygen-evolving complex, presented a negative correlation (up to 24 h) between the phosphopeptide level and the PSBR protein abundances with a 2.4-fold decrease of the phosphorylation (Additional file 3: Figure S2B) [7]. This observation suggests a specific regulation of the Oxygen-evolving complex to adjust the input of electrons. Similarly, a 2.5-fold phosphorylation decrease was observed in the proteins involved in the electron transfer chain like Pre-apoplastocyanin protein (Cre03.g182551.t1.2) (Additional file 3: Figure S2B). This protein is responsible for the flow of electrons between cytochrome b_6_f and photosystem I. Altogether, observed phosphorylation changes suggest that a “pseudo”-highlight response occurs in the first 24 h of N depletion.

### Lipid accumulation

During N depletion, lipid accumulation has been observed (Fig. 1). In contrast, proteomics data published by Valledor et al. indicated that most of the proteins related to de novo fatty acid synthesis were stable or surprisingly decreasing [7]. Hence, several studies have presented different mechanisms of TAG accumulation in lipid droplets (LDs) under N starvation: a recycling of membrane lipids into TAG, an increased de novo synthesis of TAG from acyl‐CoA, and an increased carbon flux toward glycerol‐3‐phosphate and acyl‐CoA for fatty acid synthesis [59–63]. Among the phosphoproteomics data, protein phosphorylation changes support the hypothesis that the plastid undergoes degradation (including thylakoids membrane), while total lipid content increases from 24 h on. Indeed, proteins involved in plastid vesicle formation such as plastoglobules (lipid bodies) [64] were also differentially phosphorylated. Cre07.g325736* and Cre02.g143667* are part of the fibrillin sub-family, the plastid-associated lipid protein (PAP). Fibrillin is known to associate to plastoglobules to participate in their formation [65] (Additional file 2: Table S2). In line with a degradation of some proteins related to fatty acid synthesis, decrease of an enoyl-ACP reductase (Cre06.g294950*—Ser55 and Ser57) was observed by Valledor et al. [7]. Phosphopeptides belonging to the enoyl-ACP reductase which is a component of the fatty acid synthase complex show a 3.8-fold increase in phosphorylation levels up to 77 h suggesting that the enoyl-ACP reductase phosphorylation is a regulatory mechanism of the lipid metabolism (Additional file 2: Table S2). Finally, two proteins related to lipid degradation presented differential phosphorylation pattern. One is a triacylglycerol lipase Cre17.g699100 showing a de-phosphorylation of the Ser912 during N depletion, the other one is a specific diacylglycerol lipase beta form (Cre10.g463600—T1982) with a phosphorylation pattern stable over N depletion and peaking at 77 h (Additional file 2: Table S2).

### Starch accumulation and carbohydrate metabolism

**Fig. 6:**
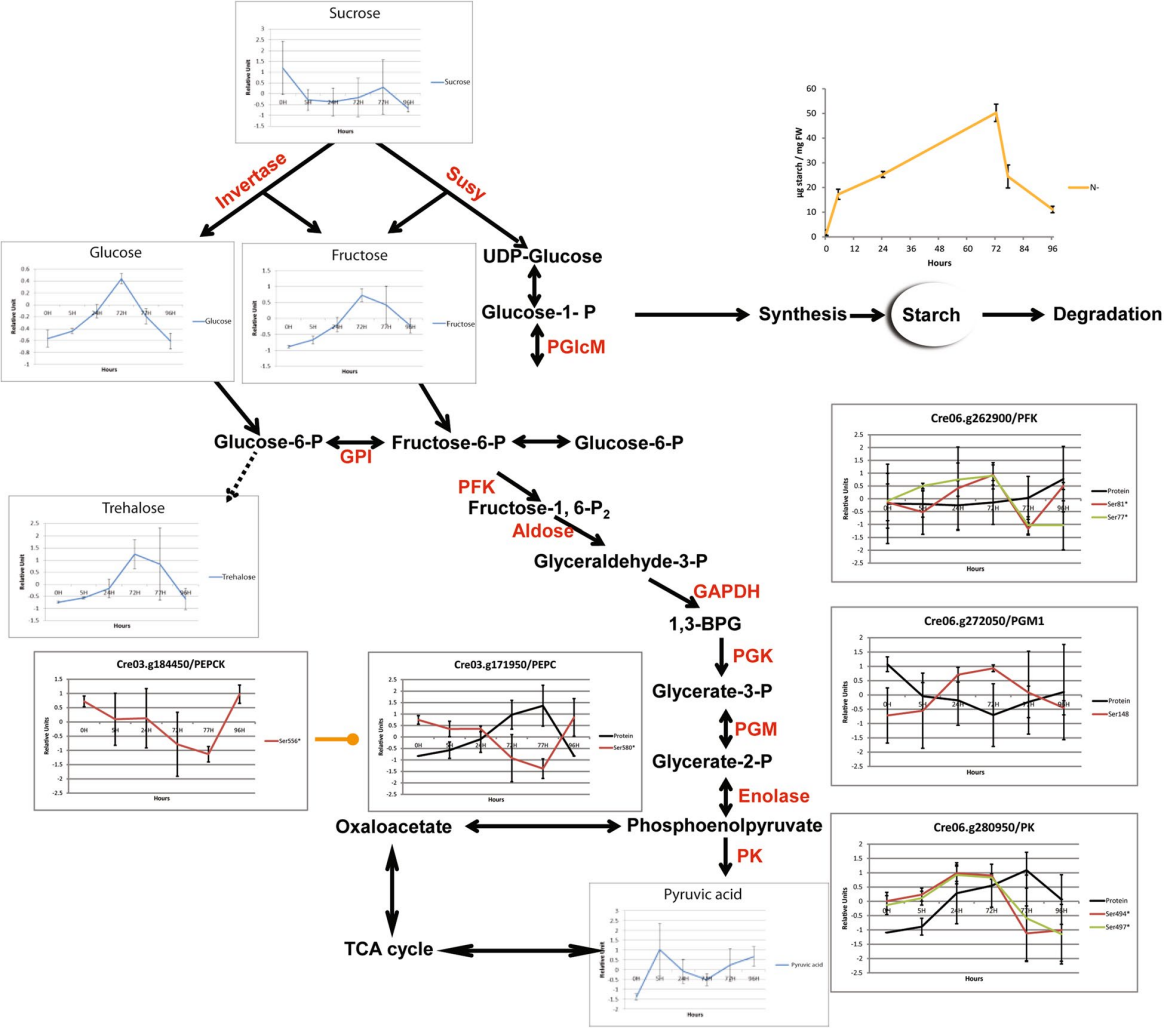
Visualization of the Phosphorylation changes identified at primary metabolism. Summary of changes in protein phosphorylation involved in the regulation of the metabolism. Data represent Z-transformed normalized abundances of metabolites, proteins, and phosphopeptides (*n* = 3 for phosphopeptides and *n* = 4 for protein level and metabolites). Metabolites are reported in blue, protein level in black, and phosphosites levels in red. Phosphosites followed by * are significant (Additional file 2: Table S2). Proteins and metabolites levels are based on data obtained previously [7]

Like lipids, starch accumulates during N depletion and undergoes quick degradation during N recovery (Fig. 1). While Valledor et al. already demonstrated that starch and glycolysis metabolism-related enzymes undergo specific regulation during nitrogen depletion and recovery [7], no information about phosphorylation changes were obtained. Quantitative in vivo phosphoproteomics analysis revealed a 4.3-fold de-phosphorylation of a starch water dikinase (Cre03.g183300—Ser39) during N depletion (Additional file 2: Table S2). Additionally, several proteins involved in glycolysis and gluconeogenesis were found to be differentially regulated at the phosphopeptide level. We identified in our dataset phosphopeptides belonging to a phosphoenolpyruvate carboxylase (PEPC) (Cre03.g171950*—Ser580), a chloroplastic phosphofructokinase (PFK) (Cre06.g262900*—Ser77 and Ser81), and a pyruvate kinase (PK) (Cre06.g280950*—Ser494 and Ser497) presenting significant differences over the experiment (Fig. 6). Both PFK and PK are important regulatory enzymes of the glycolysis since they perform irreversible steps. Their phosphosites are twofold upregulated during N depletion, and down-regulated in N replete samples (Fig. 6). On the contrary, PEPC phosphorylation presented a 2.3-fold decrease during N depletion and increased with N recovery. In mammals and yeast, PFK can be inhibited by protein kinase phosphorylation at the N-terminus [66]. *Chlamydomonas* PFK phosphorylation occurs in the N-ter segment at Ser77 and Ser81. A similar phosphosite was found for *Arabidopsis* PFK1 at Ser71 [67]. To avoid a futile cycle between PK and PEPC, it can be suggested that PK is inhibited by phosphorylation. In line with a PK inhibition and PEPC activation it was observed that PEPC activity in CAM plants is controlled through phosphorylation which increases the enzymatic activity [68–70]. PEPCK (phosphoenolpyruvate carboxylase protein kinase) is the protein responsible for the PEPC phosphorylation [68–71]. In our dataset we found that PEPCK (Cre03.g184450—Ser556) protein phosphorylation follows the same pattern as PEPC phosphorylation at Ser580 (*r* = 0.97) (Fig. 6). Glycolysis inhibition is further supported by the data obtained for Cre06.g272050*, an independent phosphoglycerate-mutase (PGM1) which catalyzes the reversible inter-conversion of 3-phosphoglycerate to 2-phosphoglycerate. Valledor et al. showed that this protein decreased threefold during N depletion, while we found that the phosphosite at Ser148 presented an inverse trend (*r* = − 0.78) with a threefold increase of the phosphorylation abundance (Fig. 6). *Arabidopsis* double iPGAM mutants with no detectable activity showed impaired vegetative plant growth and pollen development [72].

### Phosphoproteomics data suggest a specific regulation of plastidic and cytosolic ribosomal translation activity during N depletion

Regulation of translation is a key process for the metabolic adaption to varying nitrogen availability. Key factors of the translational activity regulation are the ribosomes. Previously, Valledor et al. observed a complete remodeling of the proteomics data associated with a decrease of plastidic ribosomal proteins while cytosolic ribosomal proteins were increasing during N depletion [7]. A detailed analysis revealed that chloroplast ribosomes are degraded mainly from 24 to 72 h of N depletion. Some chloroplast ribosomal proteins (RPs) such as PRPL15 (Cre14.g612450*—Ser38) presented a negative correlation between the protein and phosphorylation sites from 0 to 24 h (Additional file 3: Figure S2B). After N repletion, the protein and phosphorylation levels were synchronized and followed similar trends (Additional file 3: Figure S2B). In the case of PSRP3 (Cre02.g083950*—Ser120) and PRPL32 (Cre07.g352850*—Ser90), an approximately 2.4-fold de-phosphorylation occurred for both proteins during N depletion while the phosphorylation fully recovered after the nitrogen recovery. Connected to a potential increase in chloroplast acclimation between 0 h and 24 h, TOC75 (75kD protein of the translocon outer membrane) (Cre03.g175200*—Ser147, Thr145, and Ser344) showed a phosphorylation peak at 24 h after nitrogen depletion (Additional file 3: Figure S2B). Those data correlate with the observation of higher amounts of PSII and LHCII proteins observed from 0 to 24 h (Additional file 3: Figure S2A), and suggest that phosphorylation of plastid ribosomal proteins plays a role in plastid translational activity regulation. Similar conclusions can also be derived for the cytosolic ribosome, for which it is known that their phosphorylation allows for a fine control of translation in all eukaryotic cellular systems examined so far [73]. Concerning large cytosolic ribosomal proteins, a significant phosphosite on RPL10 at position Ser104 was found with a 4.5-fold increase during N depletion. On the other hand, RPL10 protein level decreased during the first 24 h to a stable level at 72 h (Additional file 4: Figure S3B) [7]. The role of RPL10 in chloroplast homeostasis regulation was previously hypothesized [74]. The potential function of RPL10 on plastid homeostasis is further strengthened by the dynamics of chloroplast-related proteins and chloroplast degradation. The small cytosolic ribosomal proteins RPS15, RPS25, and RPS27, showed an threefold decrease in their phosphorylation during N depletion (Additional file 4: Figure S3B). RPS6 phosphorylation at Ser245 showed a 2.5 increase during N depletion following the same trend as the protein level (Additional file 4: Figure S3B). This observation is consolidated by the constitutive phosphorylation of acidic ribosomal protein P2 at both Ser67 and Ser98 (UID521 and UID522) which is required for RPS6 assembly into the 60S subunit [75] (Additional file 4: Figure S3B).

### Eukaryotic initiation factor (EIF) phosphorylation coordinates the proteome acclimation to nitrogen depletion and recovery

**Fig. 7:**
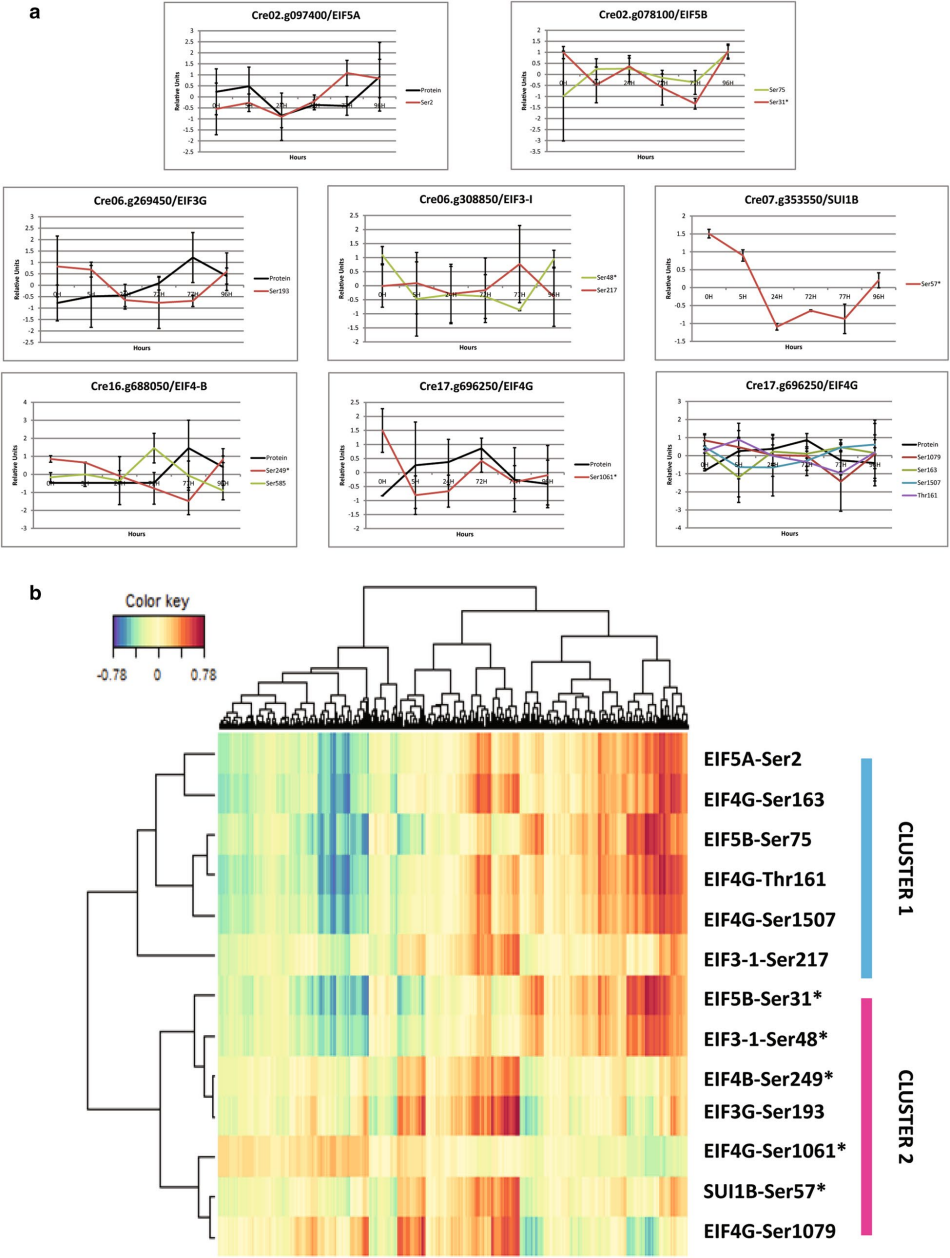
Eukaryotic initiation factors and phosphorylation changes coordinate the proteome acclimation to nitrogen availability. **a** Phosphopeptide correlation with protein levels for the proteins involved in the translational initiation. Eukaryotic initiation factors present changes in their phosphorylation level. Data represent Z-transformed normalized abundances of protein and phosphopeptides belonging to eukaryotic initiation factors (*n* = 3 for phosphopeptides and *n* = 4 for protein level). Phosphosites followed by * are significant (Additional file 2: Table S2). Protein levels are based on data obtained previously [7]. **b** SPLS analysis between the EIF phosphosites used as predictor and the proteome used as response factors. All correlations can be found in Additional file 2: Table S8

Several phosphopeptides of different eukaryotic initiation factor proteins (EIFs) showed significant variation during N depletion and recovery (see Protein interaction network Fig. 4a). In the cytosol, EIFs form a complex with ribosomes thereby fine tuning translational activity. To gain more insight of EIF control on translational activity regulation, SPLS analysis was conducted with EIF phosphopeptides to predict changes at the proteome level (Fig. 7b and Additional file 2: Table S8). Interestingly, two clusters of phosphorylation sites correlate differentially with the proteome acclimation (Fig. 7b). Cluster 1 contains EIF4B (S249, S585), EIF4G (S1061, S1079), SUI1B (S57), EIF5B (S31), EIF3-1 (S48), and EIF3G. Cluster 2 contains the phosphosites EIF4G (S161, T163, S1507), EIF5B (S75), EIF5A, and EIF3-1(S217). Accordingly it is the combination of several EIF phosphosites rather than single changes which explain the proteome adaptation to nitrogen starvation and recovery best. SPLS revealed positive correlations between proteins involved in lipid metabolism and EIF4G Ser161, and negative correlations between cluster 1 and the major lipid droplet protein (Cre09.g405500) (Additional file 2: Table S8). Among the phosphoproteins, two well-known eukaryotic factors are EIF4B and EIF4G. EIF4B protein has been found to be increasingly de-phosphorylated in response to stressful environmental changes in plant cells [76, 77]. In our data, EIF4B (Cre16.g688050) proteins also showed a differential phosphorylation pattern, at Ser249, to N availability, with a 3.4-fold decrease during N depletion followed by an increase after N repletion (Fig. 7a). On the other hand, EIF4G (Cre17.g696250*) protein is phosphorylated in the N-terminus region as we found phosphorylation sites at Thr161 and Ser163, similar to the one found in *Arabidopsis* [78]. Phosphosites were also found in the conserved MIG4 domain (Ser1061 and Ser1079) interacting with EIF3 and EIF4E in yeast [79]. Finally, EIF4G was phosphorylated in the C-terminus domain at Ser1507 which presents a negative correlation with the protein level (*r* = − 0.79) (Fig. 7a).

### Autophagy and ubiquitination pathways are regulated by protein phosphorylation during N depletion

In line with previous data indicating that N stress as well as TOR inhibition induces autophagy [35], we identified ATG13 (Cre16.g659000—Ser1201) to be differentially phosphorylated between N depletion and N recovery (Additional file 5: Figure S4B). ATG13 showed a 1.5-fold increase at Ser1201 during the N depletion and a 2.5-fold decrease after N replenishment. Autophagy is a complex process of degradation of cell components essential for cell survival upon nutrient limitation by promoting membrane recycling as well as organelle recycling [80]. Further, proteins associated to vesicular trafficking such as Vacuolar Protein Sorting (VPS) are also involved in autophagy processes [81]. Indeed VPS proteins interact with ATG proteins to promote autophagosome formation. In our phosphoproteomics data, Cre14.g628050* (VPS51/67—Ser1312) and Cre06.g310000 (VPS53—Ser820) proteins were differentially regulated during the experiment (Additional file 5: Figure S4B). The phosphorylation level of VPS51/67 increased until 24 h. The protein and phosphopeptide levels are then synchronized and increase concomitantly after N repletion. On the other hand, VPS53 showed a significant 3.2-fold decrease at the phosphosite Ser820 during N depletion, followed by a 2.7-fold increase, up to 96 h, to similar phosphorylation level as the 0 h time point (Additional file 5: Figure S4B). Other proteins related to vesicle sorting such as Cre07.g326450 (VPS5B—Ser48), which are part of the retromer complex, are also important for synchronizing Golgi activity with all the other vesicular systems [82]. The Ser48 phosphosite showed a stable phosphorylation level during N depletion followed by a de-phosphorylation after N repletion (77 h) (Additional file 5: Figure S4B).

Based on a more detailed analysis of the Valledor et al. proteome data, we observed that the proteins involved in the 26S proteasome, E3 SCF complex and E1 presented an increase over N depletion followed by a decrease after N recovery (Additional file 5: Figure S4A). On the other hand the general trend of proteases, E2 and E3 ring related proteins showed inverse trends (Additional file 5: Figure S4A). At the phosphopeptide level, the regulatory protein of the 26S proteasome complex, RPN2 (Cre17.g727950*—Ser873), showed a rapid increase in the phosphorylation level in the first 5 h of N depletion and then a down-regulation until N recovery (Additional file 5: Figure S4A). On the other hand, proteins involved in the E3 SCF FBOX complex (Cre12.g520650*—Ser26, Ser32, Ser106, Ser115) showed a higher phosphorylation level over N depletion and a down-regulation of this level during the N recovery (Additional file 5: Figure S4B). Altogether those data suggest that both autophagy and ubiquitination pathways are activated during N depletion.

## Discussion

**Fig. 8:**
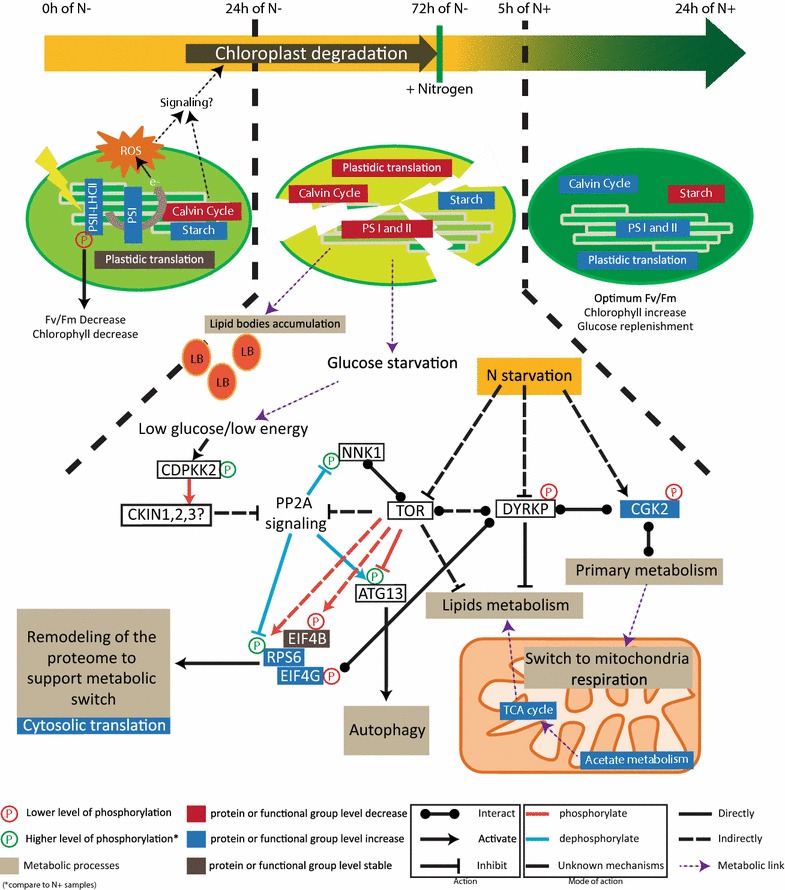
Model for regulation of nitrogen availability response. Our data as well as previous microscopy data suggest that the nitrogen depletion plays a role in chloroplast structure and metabolism [7, 18, 42]. More precisely our data support a two-phase acclimation of the chloroplast during N depletion. A first highlight-like response due to the depletion of stromal protein and supported by the accumulation of LHCII/PSII protein which leads potentially to ROS acclimation, both events could trigger a signal for chloroplast degradation as suggested in *Arabidopsis* [90]. High phosphorylation levels of CP29 at the early stage of N depletion support those data and could be implicated in the regulation of Fv/Fm [88]. The second step consists in the dismantlement of the chloroplast from 24 h on. Chloroplast dismantlement might involve the BR signaling pathway (see “Results”). Two protein kinases were shown to play a central role, CGK2 and DYRKP. They are connected to central metabolism which shows a clear switch to mitochondrial respiration (a process tightly controlled by protein phosphorylation) and protein translation (tight control by EIF phosphorylation). Data obtained for EIF4B phosphorylation are suggesting a down-regulation of the TOR complex. In this model, we propose that the higher phosphorylation levels observed for NNK1, RPS6, and ATG13 indicate a possible inhibition of the PP2A branch signaling of the TOR pathway by activation of CKIN pathway (CKIN 1, 2, and 3 are the AMPK/SnRK1 orthologs in *Chlamydomonas* as defined in [6]). This is further suggested by the higher phosphorylation level of CDPKK2, as proposed in yeast [102]. For example, the red-dashed arrow between TOR and RPS6 indicate that TOR indirectly and positively regulates RPS6 by phosphorylation. ROS is reactive oxygen species; PS is photosystem; LHCII is Light-Harvesting complex-II; e represents electron from the electron transfer chain; and LB stands for Lipid Bodies

In this study, we have confirmed the robustness and reproducibility of the experimental set-up for the analysis of nitrogen depletion and recovery initially introduced by Valledor et al. [7]. Consequently, this experimental system allows for the integration of further molecular studies into a comprehensive systemic approach to understand complex biochemical regulation of starch and lipid accumulation in the biomass/biofuel model *C. reinhardtii*. Here, we report for the first time a comprehensive dataset for the *Chlamydomonas* phosphoproteome acclimation during N depletion and recovery. We observed that 55% of the identified phosphoproteins are annotated with unknown protein function compared to only 25% in the proteomic dataset of Valledor et al. [7]. These results clearly demonstrate that especially the phosphoproteome and its related functions are rather undiscovered and future studies are needed to focus on signaling networks in *C. reinhardtii*. Integration of phosphoproteomics data with other experimental high-throughput data such as proteomics and metabolomics revealed not only a complex regulatory network of different signaling pathways involved in metabolism but also cellular and intracellular development in these single cell green algae. These results are summarized in a proposed model of *Chlamydomonas* acclimation to nitrogen depletion and recovery as shown in Fig. 8. A systemic analysis is the requirement to eventually understand the cell’s adaptation to N availability because our data suggest an interwoven multi-processes adaptation not explainable with separated processes (Fig. 8). Furthermore, we provide evidence that phosphopeptide levels are hardly interpretable without the protein level, and therefore a combined analysis between both is required. As example, the phosphorylation at the residue Thr7 on CP29, which is known to be a constitutive phosphorylation site of CP29 [58, 83], showed similar dynamics as the protein level (Additional file 6: Figure S5). Altogether our results indicate that increasing the coverage between phosphoproteomics and proteomics analysis will improve phosphoproteomics data interpretation.

### The two-phase acclimation of the chloroplast during N depletion

Recently, chloroplast degradation including thylakoid membranes was shown using electron microscopy (EM). EM data indicated that chloroplast degradation occurs mainly from 20 h on after N depletion [18, 42]. In line with EM observations, several proteins associated to thylakoid membrane formation were differentially phosphorylated in our study, such as VIPP1 (Cre13.g583550*) (Additional file 3: Figure S2B). VIPP1 protein is responsible for the formation of thylakoid structures in *Chlamydomonas* [84, 85]. Those observations are further supported by the dynamic expression level of proteins involved in plastid development and thylakoid structure, such as TIG1 and THF1 [86, 87], (Additional file 3: Figure S2B). Interestingly, the increase in some plastid ribosomal proteins as well as the phosphorylation of TOC75 protein suggests that the chloroplast and the photosystem are preparing to acclimate during the first 24 h, while chloroplast dismantling already started (Additional file 3: Figure S2B). Those observations are in line with the dynamics of functional categories involved in photosynthesis. Indeed, an increase of proteins belonging to PSI and LHCII from 0 to 24 h was observed (Additional file 3: Figure S2A and Fig. 8). After 24 h of N starvation, all these proteins show decreased levels. This observation was surprising since Fv/Fm showed a rapid decrease in PSII intactness occurring in the first 5 h of N depletion (Figs. 1d and 8). It was proposed that PSII intactness is regulated by CP29 phosphorylation in monocots [88]. Interestingly, the phosphoproteomics data point out a putative “pseudo”-highlight stress response. Indeed, it was shown that under highlight stress CP29 threonine at position 11, 17, 18, 27, 33 showed higher phosphorylation level [58]. Here, we observed that in the first 24 h of N depletion CP29 phosphorylation on those sites was increasing (Additional file 3: Figure S2B) suggesting a response in the first 24 h of N depletion similar to that in highlight. Also, PSBR phosphorylation suggest that PSBR is required for the stable binding of LHCSR3 to PSII–LHCII super complexes as it is required for efficient energy-dependent quenching under continuous highlight [89]. Such highlight stress-like response can be due to an inhibition of the stromal reactions, mainly represented by the Calvin cycle depletion (Additional file 3: Figure S2A and Fig. 8). Eventually, we propose that two signaling mechanisms are involved in chloroplast degradation. The first one would involve an activation of the brassinosteroid pathway which is known in *Arabidopsis* to inhibit chloroplast development [55]. The second signaling pathway comprises a mechanism for plastid degradation through autophagy processes as it has been proposed in *Arabidopsis* [90]. The signal is triggered by both stromal protein depletion and simultaneous ROS accumulation in plastids. In our case, results suggest that a “pseudo”-highlight stress response in combination with the stromal protein depletion occurring in the first 24 h could lead to ROS production thereby inducing the plastid degradation processes (Fig. 8). It should also be noted that lipid accumulation correlates with chloroplast degradation [18, 42] providing further insight on the intricate regulation between plastid membrane recycling and lipid accumulation.

### Chloroplast status is connected with energy metabolism changes

Evidence that plastid degradation is affecting the glycolytic pathway was also found at the metabolite level (see Fig. 6) [7]. Measured sugars such as glucose, fructose, trehalose showed a higher accumulation rate from 24 to 72 h compared to 0–24 h [7]. Those higher rates of sugar accumulation correlate with the decrease in malate and fumarate after 24 h suggesting that TCA cycle is not anymore fueled by glycolysis [7]. This finding can be correlated with the increased phosphorylation level on the PFK, PK, and PGAM enzymes during N depletion. PFK regulation is a potential target for biofuel application since evidence in plant and microalgae exists on the role of PFK and F6BP in carbon partitioning [91, 92]. Those data indicate that degradation of the plastid leads to a lower glycolytic activity as previously indicated [7, 63]. Intriguingly, these results highlight that on the long-term N depletion the cells have to deal with glucose/energy stress as suggested in our model (Fig. 8). The fact that cell division still occurs in the very early phase of N depletion might indicate a metabolic switch from plastid activity to mitochondrial respiration in order to support the minimal amount of cell activity. Accumulation of proteins involved in acetate degradation such as ACK2 and PAT1 occurred in parallel to an increase of proteins related to mitochondrial oxidative phosphorylation and TCA cycle and support an increase of the mitochondrial activity [7]. Therefore, acetate incorporation is used to fuel mitochondrial activity [7].

### Energy signaling pathways coordinate N stress response

In line with a low energy level induced by nitrogen depletion, the results for signaling protein kinases such as DYRKP and CDPKK2 point to an activation of energy saving pathways such as AMPK and a down-regulation of the antagonist TOR (Fig. 8). First of all it should be noted that the relative amount of cytosolic RPs is increasing compared to the plastid RPs as it was observed in Arabidopsis TOR RNAi lines [93]. Additionally, several phosphoproteins related to TOR signaling pathway were identified: EIF4B, RPS6, ATG13, and NNK1, an interactor of the TOR complex in yeast [94]. Evidence for TOR inactivation is given by the decreasing phosphorylation level of the TOR-target EIF4B during N depletion, while the phosphorylation level was reverted by N re-addition. However, other data obtained for RPS6, ATG13, and NNK1 are contradictory for a TOR inhibition during N depletion. RPS6 activity is controlled by the TOR pathway which triggers RPS6 phosphorylation via S6K activity [73]. Homologs of TOR and S6K exist in plants and *Chlamydomonas* [27, 95, 96], and RPS6 has indeed been found to be phosphorylated in Arabidopsis most probably downstream of an AMPK–TOR antagonism [29]. Here, we have also detected RPS6 phosphorylation (Additional file 4: Figure S3B). In *Chlamydomonas*, it is hypothesized that N availability is a direct regulator of translation via TOR signaling [34]. Therefore, it could be assumed that RPS6 phosphorylation is a direct readout of TOR activity, as suggested in Arabidopsis and mammals [73, 93]. However, RPS6 showed increasing phosphorylation levels and an increasing protein amount during N depletion. Several aspects need further investigation: (i) how is the TOR–S6K–RPS6 pathway regulated in *Chlamydomonas reinhardtii* [97], and (ii) is RPS6 differentially multiphosphorylated and (iii) is RPS6 differentially distributed in subcellular compartments. ATG13 is part of the ATG1 kinase complex which promotes autophagy, through its association with ATG8 and VPS proteins [98], while it is negatively regulated by TOR complex 1. In yeast, under nutrient-sufficient conditions ATG13 is hyper-phosphorylated. Under nutrient depletion TORC1 is inhibited and ATG13 is no longer phosphorylated [99–101]. We found that ATG13 phosphorylation is increasing during N depletion and decreasing during N recovery, showing therefore opposite results to an expected TOR inhibition (Additional file 5: Figure S4B). Finally NNK1 was found to be more phosphorylated in our conditions while it was found to be de-phosphorylated under N depletion or rapamycin treatment in yeast [102]. However, in yeast, under N depletion plus glucose starvation the PP2A branch signaling of the TOR pathway was inhibited triggering a higher phosphorylation level of NNK1 [102]. Similarly, in yeast, PP2A has been found to antagonize ATG13 phosphorylation by TORC1 to activate autophagy [103]. In plants PP2A has been found to regulate RPS6 phosphorylation [96]. The regulatory network involving TOR deactivation and PP2A activity needs to be further addressed by additional studies in future. A model is proposed summarizing these discussions in Fig. 8.

### DYRKP—a central coordinator of lipid metabolism

The accumulation of lipids and starch is a highly reversible process after re-addition of nitrogen to the culture (see Fig. 1e, f) indicating the plasticity of the molecular processes to preserve or provide chemical energy depending on the environmental conditions. Phosphoproteomics data revealed dynamic in vivo protein phosphorylation of enzymes involved in lipid metabolism showing clear evidence that protein phosphorylation plays a role for the reprogramming of lipid metabolism and accumulation. One of the central kinases identified in the protein–protein interaction network (Fig. 4a) is DYRKP which was found as a negative regulator of starch and lipid accumulation [26]. DYRKP protein was also connected to CGK2 and EIF4G (Fig. 4a). In our study, particularly the phosphorylation sites on EIF4G showed interesting dynamics. On the one hand, the phosphorylation levels at Ser1061 and Ser1079 in the MIG4 domain of EIF4G are grouped together in cluster 1 (Fig. 7b). On the other hand, the phosphosites at the N-terminus and at position S1507 belong to cluster 2 (Fig. 2b). These results suggest that EIF4G phosphorylation sites are reciprocally regulated based on N availability. EIF4G phosphorylation at position Ser1061 correlates positively with proteins involved in lipid metabolism (Additional file 2: Table S8) such as the major lipid droplet protein (Cre09.g405500) (Additional file 2: Table S8) [104]. EIF4G and DYRKP interaction could explain how DYRKP controls oil accumulation (Fig. 4a). As master regulators of the translational activity, both EIF4B and EIF4G appeared to be interesting targets for molecular engineering.

## Conclusion

Microalgae are economically important as potential sources for biofuel production, especially because they do not compete with nutritional crops for arable land. As a photoautotrophic/mixotrophic model organism, *C. reinhardtii* is a suitable candidate for studying TAG and starch accumulation during N depletion and recovery. We report for the first time a comprehensive dataset for *Chlamydomonas* phosphoproteome acclimation during N depletion and recovery. The analysis demonstrated that chloroplast structure and activity, sugar, and lipid metabolism as well as protein turnover show significant and reversible changes at the phosphoprotein level in response to N depletion and recovery. Integration of phenotypic parameters with protein phosphorylation changes revealed the central involvement of protein kinases such as DYRKP, CGK2, and CDPKK2 in growth as well as starch and lipid accumulation (Fig. 8). Our data suggest a two-phase adaptation of the chloroplast during N depletion: a short-term acclimation (from 0 to 24 h), to “pseudo-highlight”—stress most probably due to the depletion of Calvin cycle proteins, and a long-term acclimation which involves chloroplast and thylakoid membrane degradation (Fig. 8). Chloroplast degradation leads to a metabolic switch toward mitochondria supported by metabolism fine tuning, especially at the glycolysis level (Fig. 8). Results for signaling protein kinases such as DYRKP and CDPKK2 point to an activation of energy saving pathways such as CKIN (AMPK in *Chlamydomonas* [6]) and a down-regulation of the antagonist TOR complex. Inhibition of TOR complex is supported by the EIF4B phosphorylation level. However, data obtained for other TOR targets such as ATG13, RPS6, and NNK1 indicate that a more complex regulatory mechanism is active potentially involving TOR-complex regulatory PP2A branch signaling in *Chlamydomonas* (Fig. 8). Our study validates the reproducibility of the experimental set-up which provides a frame toward improving our understanding of signaling pathways occurring during N depletion and replenishment in *Chlamydomonas*. It also allows a more precise study of the metabolic bottlenecks for N availability acclimation, such as the glycolytic pathway. In this context, energy sensing and signaling function in *Chlamydomonas*, as well as the chloroplast degradation process in long-term adaptation and their potential link with lipid accumulation and energy metabolism are of particular interest.

## Methods

### Plants material and growth conditions

*Chlamydomonas reinhardtii* CC-503 *cw92*, mt+, agg1+, *nit1*, *nit2* cultures were grown in HEPES–Acetate–Phosphate medium supplemented with 7 mM NH_4_Cl (HAP  +  N; TAP medium in which Tris was replaced by 5 mM HEPES) at 25 °C with shaking (120 rpm) in a 16:8 light:dark photoperiod (85 μmol m-2 s-1; Sylvania Grolux lamps). To start the experiments, cultures were pelleted down by centrifugation at the end of the night, washed two times with HAP-N medium, and re-suspended in HAP-N media (NH_4_Cl was replaced by 7 mM KCl) to a final density of 1–3  ×  10^5^ cells mL^−1^. From this point, cells were cultivated under continuous light. Cells were sampled at 0, 5, 24, and 72 h time points after N depletion. After the 72 h time point, NH_4_Cl was added to the HAP-N cultures to a final concentration of 7 mM to start the recovery phase. The cultures were once again sampled at 5 h (77 h) and 24 h (96 h) after the addition of N.

### Physiological measurements

Growth was measured through the use of two techniques: counting the number of cells and/or measuring the fresh weight of a specific volume of the culture. Cell number was measured by using a Thoma hemocytometer and a light microscope (Nikon eclipse S5i). Fresh weight was acquired through gravitometric measurements. Starch was measured by using an enzymatic assay as described by Staudinger [105]. Total lipid content was measured as described by [7]. Chlorophyll content was monitored as described by [106]. The photosynthetic rate was measured with an imaging/pulse-amplitude modulation fluorimeter (OS1-FL, Opti-Sciences).

### Protein extraction and phosphopeptide enrichments

Total protein was extracted from *Chlamydomonas* cell pellets using 500 μl of extraction buffer (100 mM Tris–HCl pH 8.0; 5% SDS, 10% glycerol; 1.4 M sucrose; 10 mM DTT; supplemented with protease and phosphatase inhibitor cocktails as indicated by the supplier (Roche, Cat. No. 05 892 791 001 and Cat. No. 04 906 837 001)). The samples were incubated at room temperature for 5 min. Immediately after the extraction, 300 µl Roti-phenol was added to the samples, which were then vortexed and incubated during 5 min before centrifugation at 21,000×*g* for 5 min at room temperature. The supernatant was carefully transferred to a new tube. Phenol extraction was repeated a second time. The phenol fractions were pooled together and counterextracted with 300 μl of extraction buffer and centrifuged at 21,000×*g* for 5 min at room temperature. The supernatant was carefully transferred to a new tube. Protein precipitation was performed by mixing the phenol fraction with 2.5 volumes of 0.1 M ammonium acetate in methanol. After a 16-h incubation period at − 20 °C, the samples were centrifuged for 5 min at 5000×*g*. The protein pellets were washed twice with 0.1 M ammonium acetate, one time with acetone, and air dried at room temperature. The protein pellets were dissolved in 8 M urea/100 mM ammonium bicarbonate (AmBic) supplemented with protease and phosphatase inhibitor cocktails as indicated by the supplier (Roche, Cat. No. 05 892 791 001 and Cat. No. 04 906 837 001). Protein concentration was determined using the Bio-Rad Bradford Assay with BSA as a standard. 100 μg of total protein per sample was first reduced with dithiothreitol (DTT) at concentration of 5 mM at 37 °C for 45 min. Cysteine residues were alkylated with 10 mM iodoacetamide (IAA) in darkness at room temperature (RT) for 60 min. Alkylation was stopped by increasing DTT concentration to 10 mM and incubating the samples in the dark at RT for 15 min. Then the urea concentration was diluted to 2 M with 50 mM AmBic/10% acetonitrile (ACN). CaCl2 was added to a final concentration of 2 mM. Trypsin digestion (Poroszyme immobilized trypsin; 5:100 v:w) was performed at 37 °C overnight. Protein digests were desalted with C18 solid phase extraction (SPE) (Agilent Technologies, Santa Clara, USA) and carbon graphite SPE as described by [107]. After both SPEs, the corresponding eluates were pooled and dried in a vacuum concentrator. Phosphopeptide enrichment was performed using 5 mg of TiO2 (Glygen Corp.) as described previously [29, 38] and dried in a vacuum concentrator.

### LC–MS for phosphoproteomics

Phosphopeptide pellets were resolved in 10 µl of 5% (v/v) ACN and 0.5% (v/v) formic acid (FA). 5 µl of the mixture was separated on an EASY-Spray PepMap RSLC 75 μm × 50 cm column (Thermo Fisher Scientific Inc., Waltham, USA). Peptides were eluted using a 240-min linear gradient from 2 to 40% of mobile phase B (mobile phase A: 0.1% [v/v] formic acid (FA) in water; mobile phase B: 0.1% [v/v] FA in 90% [v/v] ACN) with 300 nl/min flow rate generated with an UltiMate 3000 RSLCnano system.

The peptides were measured with a LTQ-Orbitrap Elite (Thermo) using the following mass analyzer settings: ion transfer capillary temperature 275 °C, full scan range 350–1800 m/z, and FTMS resolution 120,000. Each FTMS full scan was followed by up to ten data-dependent (DDA) CID tandem mass spectra (MS/MS spectra) in the linear triple quadrupole (LTQ) mass analyzer. Dynamic exclusion was enabled using list size 500 m/z values with exclusion width ± 10 ppm for 60 s. Charge state screening was enabled and unassigned and + 1 charged ions were excluded from MS/MS acquisitions. For injection control, automatic gain control (AGC) for full scan acquisition in the Orbitrap was set to 5 × 10^5^ ion population, and the maximum injection time (max IT) was set to 200 ms. Orbitrap online calibration using internal lock mass calibration on m/z 371.10123 from polydimethylcyclosiloxane was used. Multistage activation was enabled with neural losses of 24.49, 32.66, 48.999, 97.97, 195.94, and 293.91 Da for the ten most intense precursor ions. Prediction of ion injection time was enabled and the trap was set to gather 5 × 10^3^ ions for up to 50 ms.

### Data analysis and statistics

For peptide identification against the *Chlamydomonas* database, a map for the proteome of the JGI_236 version was specifically created using Mercator (http://MapMan.gabipd.org/web/guest/app/mercator) [108]. For phosphorylation site mapping as well as phosphopeptide quantification, MaxQuant 1.5 (http://www.maxquant.org) and Andromeda search algorithm were used [109, 110]. Phosphopeptide identification was performed using the following settings: Mass tolerance for precursor was set to 5 ppm and for fragment masses up to 0.8 Da. The maximum FDR was set to 0.01%. Three missed cleavages were allowed. The dynamic modifications allowed were phosphorylation (STY), methionine oxidation (M), and protein N-terminal acetylation. The fixed modification allowed was Carbamidomethyl (C). Quantification was done at peptide level. Further data processing was done with the Perseus 1.5 software. Phosphoproteomics data were log2-transformed, and the following filtered steps were applied: only phosphopeptides belonging to category I (localization probability > 0.75 and score difference > 5) [111]. Additionally, phosphopeptides were accounted for quantification only if they were present in more than 50% of the overall samples. Data were normalized to the median of each sample and the missing values were replaced with random numbers drawn from normal distribution of each sample. *Z*-scores (zero mean, unit variance) were calculated for relative protein and phosphopeptide abundance for subsequent uni- and multi-variates analysis. One-way analysis of variance (ANOVA) and Student’s *t* test were performed with Perseus 1.5 software. PCA was performed within the numerical software environment Matlab® (V8.4.0 R2014b; http://www.mathworks.com) and the toolbox COVAIN [112] and hierarchical clustering with MeV [113, 114]. Motif-x analysis was used to identify phosphorylation motifs that were over represented in our dataset (http://motif-x.med.harvard.edu/motif-x.html) [45]. The analysis was conducted on peptides which significantly changed in the one-way ANOVA analysis. Two searches were performed against a serine residue or a threonine residue as central position. *Chlamydomonas* JGI_236 version was used as background proteome. Further details have been provided in the figure legend (Fig. 5a). Protein–protein interaction networks were created using the STRING database for Known and Predicted Protein–Protein Interactions with the standard setting (http://string-db.org/) [40]. The R software (The R Project for Statistical Computing; http://www.r-project.org/) was used to calculate SPLS analysis. Finally SPLS networks were visualized with Cytoscape v3.4.0 (http://www.cytoscape.org/) [115].

## Additional files

**Additional file 1: Figure S1.** Additional data on Chlorophyll measurement. (A) Chlorophyll [a+b] concentration by milliliter during nitrogen depletion (0 h to 72 h) and N repletion (72 h to 96 h). (B) Ratio between Chlorophyll a and Chlorophyll b (*p* < 0,05 = *; *p* < 0,001 = ***).

**Additional file 2: Table S1.** List of the 1227 quantified phosphopeptides in the whole cell for each samples. Phosphopeptides abundance was quantified based on MS1 intensity in Maxquant (see “Methods”). Data were log2 transformed and a Zero-mean normalization was applied. Percentage of coverage and number of unique peptides used for identification and score are indicated. MapMan bins were manually curated. **Table S2.** List of the 470 phosphopeptides significantly changed in the one-way ANOVA (p-value < 0.05). The mean abundance ± SD for each sampling time are indicated. **Table S3.** List of the 125 proteins identified in both proteomics and phosphoproteomics approach. For each protein the corresponding phosphopeptide(s) were added (total of 276 phosphopeptides) and coefficients of correlation were calculated. Proteomics data were zero-mean normalized. **Table S4.** PCA variable loadings of the phosphopeptides dataset. **Table S5.** Dataset for the Hierarchical clustering, based on the Mapman bin **Table S6.** sPLS analysis correlations of the integrated dataset employing phosphopeptides as predictive variables and physiological measurements as responsive variables. **Table S7.** Extraction of the network characteristics from STRING database (https://string-db.org/). **Table S8.** sPLS analysis correlations of the integrated dataset employing Eukaryotic phosphorylations sites as predictive variables and proteomics [7] data measurements as responsive variables. **Table S9.** list of protein sequences used for STRING analysis in Fig. 5a.

**Additional file 3: Figure S2.** Changes in functional group as well as in phosphorylations level related to photosynthesis (A) Mean of the proteins expression level belonging to sub-functional categories of photosynthetic related protein [7]. (B) Data represents Z-transformed normalized abundances of protein and phosphopeptides belonging to photosynthesis (n = 3 for phosphopeptides and n = 4 for protein level), Phosphosites followed by * are significant (Additional file 2: Table S2). Protein levels are based on data obtained previously [7].

**Additional file 4: Figure S3.** Changes in functional group as well as in phosphorylations level related to Protein synthesis (A) Mean of the proteins expression level belonging to a sub-functional category of protein synthesis related protein [7]. (B) Protein level and phosphosites level from specific protein belonging to protein synthesis category. Data represents Z-transformed normalized abundances of protein and phosphopeptides (n = 3 for phosphopeptides and n = 4 for protein level), phosphosites followed by * are significant (Additional file 2: Table S2).

**Additional file 5: Figure S4.** Changes in functional group as well as in phosphorylations level related to protein degradation (A) Mean of the proteins expression level belonging to a sub-functional category of protein degradation related protein [7]. (B) Protein level and phosphosites level from specific protein belonging to protein degradation category. Data represents Z-transformed normalized abundances of protein and phosphopeptides (n = 3 for phosphopeptides and n = 4 for protein level), phosphosites followed by * are significant (Additional file 2: Table S2).

**Additional file 6: Figure S5.** Dynamic of Threonine 7 phosphorylation level from CP29. CP29 Protein level and CP29-thr7 phosphosite level was plot together. Data represents Z-transformed normalized abundances of protein and phosphopeptides (n = 3 for phosphopeptides and n = 4 for protein level).

## References

[1] Mata TM, Martins AA, Caetano NS (2010). Microalgae for biodiesel production and other applications: a review. Renew Sust Energ Rev.

[2] Georgianna DR, Mayfield SP (2012). Exploiting diversity and synthetic biology for the production of algal biofuels. Nature.

[3] Hu Q, Sommerfeld M, Jarvis E, Ghirardi M, Posewitz M, Seibert M, Darzins A (2008). Microalgal triacylglycerols as feedstocks for biofuel production: perspectives and advances. Plant J.

[4] Guschina IA, Harwood JL (2006). Lipids and lipid metabolism in eukaryotic algae. Prog Lipid Res.

[5] Weckwerth W (2011). Green systems biology—from single genomes, proteomes and metabolomes to ecosystems research and biotechnology. J Proteomics.

[6] Valledor L, Furuhashi T, Hanak AM, Weckwerth W (2013). Systemic cold stress adaptation of *Chlamydomonas reinhardtii*. Mol Cell Proteomics.

[7] Valledor L, Furuhashi T, Recuenco-Munoz L, Wienkoop S, Weckwerth W (2014). System-level network analysis of nitrogen starvation and recovery in *Chlamydomonas reinhardtii* reveals potential new targets for increased lipid accumulation. Biotechnol Biofuels.

[8] Wienkoop S, Weiss J, May P, Kempa S, Irgang S, Recuenco-Munoz L, Pietzke M, Schwemmer T, Rupprecht J, Egelhofer V (2010). Targeted proteomics for *Chlamydomonas reinhardtii* combined with rapid subcellular protein fractionation, metabolomics and metabolic flux analyses. Mol BioSyst.

[9] Kempa S, Hummel J, Schwemmer T, Pietzke M, Strehmel N, Wienkoop S, Kopka J, Weckwerth W (2009). An automated GCxGC-TOF-MS protocol for batch-wise extraction and alignment of mass isotopomer matrixes from differential 13C-labelling experiments: a case study for photoautotrophic-mixotrophic grown *Chlamydomonas reinhardtii* cells. J Basic Microbiol.

[10] May P, Wienkoop S, Kempa S, Usadel B, Christian N, Rupprecht J, Weiss J, Recuenco-Munoz L, Ebenhoh O, Weckwerth W (2008). Metabolomics- and proteomics-assisted genome annotation and analysis of the draft metabolic network of *Chlamydomonas reinhardtii*. Genetics.

[11] Valledor L, Escandon M, Meijon M, Nukarinen E, Canal MJ, Weckwerth W (2014). A universal protocol for the combined isolation of metabolites, DNA, long RNAs, small RNAs, and proteins from plants and microorganisms. Plant J.

[12] Valledor L, Recuenco-Munoz L, Egelhofer V, Wienkoop S, Weckwerth W (2012). The different proteomes of *Chlamydomonas reinhardtii*. J Proteom.

[13] Merchant SS, Prochnik SE, Vallon O, Harris EH, Karpowicz SJ, Witman GB, Terry A, Salamov A, Fritz-Laylin LK, Marechal-Drouard L (2007). The *Chlamydomonas* genome reveals the evolution of key animal and plant functions. Science.

[14] Mussgnug JH (2015). Genetic tools and techniques for *Chlamydomonas reinhardtii*. Appl Microbiol Biotechnol.

[15] Wijffels RH, Barbosa MJ (2010). An outlook on microalgal biofuels. Science.

[16] Cakmak T, Angun P, Demiray YE, Ozkan AD, Elibol Z, Tekinay T (2012). Differential effects of nitrogen and sulfur deprivation on growth and biodiesel feedstock production of *Chlamydomonas reinhardtii*. Biotechnol Bioeng.

[17] Siaut M, Cuine S, Cagnon C, Fessler B, Nguyen M, Carrier P, Beyly A, Beisson F, Triantaphylides C, Li-Beisson Y (2011). Oil accumulation in the model green alga *Chlamydomonas reinhardtii*: characterization, variability between common laboratory strains and relationship with starch reserves. BMC Biotechnol.

[18] Preininger E, Kosa A, Lorincz ZS, Nyitrai P, Simon J, Boddi B, Keresztes A, Gyurjan I (2015). Structural and functional changes in the photosynthetic apparatus of *Chlamydomonas reinhardtii* during nitrogen deprivation and replenishment. Photosynthetica.

[19] Wase N, Black PN, Stanley BA, DiRusso CC (2014). Integrated quantitative analysis of nitrogen stress response in *Chlamydomonas reinhardtii* using metabolite and protein profiling. J Proteome Res.

[20] Schmollinger S, Muhlhaus T, Boyle NR, Blaby IK, Casero D, Mettler T, Moseley JL, Kropat J, Sommer F, Strenkert D (2014). Nitrogen-sparing mechanisms in *chlamydomonas* affect the transcriptome, the proteome, and photosynthetic metabolism. Plant Cell.

[21] Guarnieri MT, Nag A, Yang S, Pienkos PT (2013). Proteomic analysis of *Chlorella vulgaris*: potential targets for enhanced lipid accumulation. J Proteom.

[22] Wang X, Bian Y, Cheng K, Zou H, Sun SS, He JX (2012). A comprehensive differential proteomic study of nitrate deprivation in Arabidopsis reveals complex regulatory networks of plant nitrogen responses. J Proteome Res.

[23] Prabakaran S, Lippens G, Steen H, Gunawardena J (2012). Post-translational modification: nature’s escape from genetic imprisonment and the basis for dynamic information encoding. Wiley Interdiscip Rev Syst Biol Med.

[24] Goold HD, Nguyen HM, Kong F, Beyly-Adriano A, Legeret B, Billon E, Cuine S, Beisson F, Peltier G, Li-Beisson Y (2016). Whole genome re-sequencing identifies a quantitative trait locus repressing carbon reserve accumulation during optimal growth in *Chlamydomonas reinhardtii*. Sci Rep.

[25] Kajikawa M, Sawaragi Y, Shinkawa H, Yamano T, Ando A, Kato M, Hirono M, Sato N, Fukuzawa H (2015). Algal dual-specificity tyrosine phosphorylation-regulated kinase, triacylglycerol accumulation regulator1, regulates accumulation of triacylglycerol in nitrogen or sulfur deficiency. Plant Physiol.

[26] Schulz-Raffelt M, Chochois V, Auroy P, Cuine S, Billon E, Dauvillee D, Li-Beisson Y, Peltier G (2016). Hyper-accumulation of starch and oil in a *Chlamydomonas* mutant affected in a plant-specific DYRK kinase. Biotechnol Biofuels.

[27] Roustan V, Jain A, Teige M, Ebersberger I, Weckwerth W (2016). An evolutionary perspective of AMPK-TOR signaling in the three domains of life. J Exp Bot.

[28] Coello P, Hey SJ, Halford NG (2011). The sucrose non-fermenting-1-related (SnRK) family of protein kinases: potential for manipulation to improve stress tolerance and increase yield. J Exp Bot.

[29] Nukarinen E, Nagele T, Pedrotti L, Wurzinger B, Mair A, Landgraf R, Bornke F, Hanson J, Teige M, Baena-Gonzalez E (2016). Quantitative phosphoproteomics reveals the role of the AMPK plant ortholog SnRK1 as a metabolic master regulator under energy deprivation. Sci Rep.

[30] Robaglia C, Thomas M, Meyer C (2012). Sensing nutrient and energy status by SnRK1 and TOR kinases. Curr Opin Plant Biol.

[31] Gwinn DM, Shackelford DB, Egan DF, Mihaylova MM, Mery A, Vasquez DS, Turk BE, Shaw RJ (2008). AMPK phosphorylation of raptor mediates a metabolic checkpoint. Mol Cell.

[32] Xiong Y, Sheen J (2015). Novel links in the plant TOR kinase signaling network. Curr Opin Plant Biol.

[33] Caldana C, Li Y, Leisse A, Zhang Y, Bartholomaeus L, Fernie AR, Willmitzer L, Giavalisco P (2013). Systemic analysis of inducible target of rapamycin mutants reveal a general metabolic switch controlling growth in *Arabidopsis thaliana*. Plant J.

[34] Imamura S, Kawase Y, Kobayashi I, Shimojima M, Ohta H, Tanaka K (2016). TOR (target of rapamycin) is a key regulator of triacylglycerol accumulation in microalgae. Plant Signal Behav.

[35] Perez-Perez ME, Florencio FJ, Crespo JL (2010). Inhibition of target of rapamycin signaling and stress activate autophagy in *Chlamydomonas reinhardtii*. Plant Physiol.

[36] do Lee Y, Fiehn O (2013). Metabolomic response of *Chlamydomonas reinhardtii* to the inhibition of target of rapamycin (TOR) by rapamycin. J Microbiol Biotechnol.

[37] Perez-Perez ME, Lemaire SD, Crespo JL (2016). Control of autophagy in *Chlamydomonas* is mediated through redox-dependent inactivation of the ATG4 protease. Plant Physiol.

[38] Chen Y, Hoehenwarter W, Weckwerth W (2010). Comparative analysis of phytohormone-responsive phosphoproteins in *Arabidopsis thaliana* using TiO2-phosphopeptide enrichment and mass accuracy precursor alignment. Plant J Cell Mol Biol.

[39] Hoehenwarter W, Thomas M, Nukarinen E, Egelhofer V, Rohrig H, Weckwerth W, Conrath U, Beckers GJ (2013). Identification of novel in vivo MAP kinase substrates in *Arabidopsis thaliana* through use of tandem metal oxide affinity chromatography. Mol Cell Proteom.

[40] von Mering C, Jensen LJ, Snel B, Hooper SD, Krupp M, Foglierini M, Jouffre N, Huynen MA, Bork P (2005). STRING: known and predicted protein-protein associations, integrated and transferred across organisms. Nucleic Acids Res.

[41] Li Y, Han D, Hu G, Sommerfeld M, Hu Q (2010). Inhibition of starch synthesis results in overproduction of lipids in *Chlamydomonas reinhardtii*. Biotechnol Bioeng.

[42] Juergens MT, Deshpande RR, Lucker BF, Park JJ, Wang H, Gargouri M, Holguin FO, Disbrow B, Schaub T, Skepper JN (2015). The regulation of photosynthetic structure and function during nitrogen deprivation in *Chlamydomonas reinhardtii*. Plant Physiol.

[43] Wolschin F, Wienkoop S, Weckwerth W (2005). Enrichment of phosphorylated proteins and peptides from complex mixtures using metal oxide/hydroxide affinity chromatography (MOAC). Proteomics.

[44] Weckwerth W (2008). Integration of metabolomics and proteomics in molecular plant physiology–coping with the complexity by data-dimensionality reduction. Physiol Plant.

[45] Schwartz D, Gygi SP (2005). An iterative statistical approach to the identification of protein phosphorylation motifs from large-scale data sets. Nat Biotechnol.

[46] Hoehenwarter W, Thomas M, Nukarinen E, Egelhofer V, Röhrig H, Weckwerth W, Conrath U, Beckers GJM (2013). Identification of novel in vivo MAP kinase substrates in *Arabidopsis thaliana* through use of tandem metal oxide affinity chromatography. Mol Cell Proteomics.

[47] Rodriguez MC, Petersen M, Mundy J (2010). Mitogen-activated protein kinase signaling in plants. Annu Rev Plant Biol.

[48] Braun AP, Schulman H (1995). The multifunctional calcium calmodulin-dependent protein-kinase—from form to function. Annu Rev Physiol.

[49] Roach PJ (1991). Multisite and hierarchal protein phosphorylation. J Biol Chem.

[50] Kuenzel EA, Mulligan JA, Sommercorn J, Krebs EG (1987). Substrate-specificity determinants for casein kinase-ii as deduced from studies with synthetic peptides. J Biol Chem.

[51] Shen W, Reyes MI, Hanley-Bowdoin L (2009). Arabidopsis protein kinases GRIK1 and GRIK2 specifically activate SnRK1 by phosphorylating its activation loop. Plant Physiol.

[52] Wang Q, Pan J, Snell WJ (2006). Intraflagellar transport particles participate directly in cilium-generated signaling in *Chlamydomonas*. Cell.

[53] Wilson NF, Iyer JK, Buchheim JA, Meek W (2008). Regulation of flagellar length in *Chlamydomonas*. Semin Cell Dev Biol.

[54] Luo MN, Cao MQ, Kan YN, Li GH, Snell W, Pan JM (2011). The phosphorylation state of an aurora-like kinase marks the length of growing flagella in *Chlamydomonas*. Curr Biol.

[55] Yu X, Li L, Zola J, Aluru M, Ye H, Foudree A, Guo H, Anderson S, Aluru S, Liu P (2011). A brassinosteroid transcriptional network revealed by genome-wide identification of BESI target genes in Arabidopsis thaliana. Plant J.

[56] Wang ZY, Nakano T, Gendron J, He JX, Chen M, Vafeados D, Yang YL, Fujioka S, Yoshida S, Asami T (2002). Nuclear-localized BZR1 mediates brassinosteroid-induced growth and feedback suppression of brassinosteroid biosynthesis. Dev Cell.

[57] Ceserani T, Trofka A, Gandotra N, Nelson T (2009). VH1/BRL2 receptor-like kinase interacts with vascular-specific adaptor proteins VIT and VIK to influence leaf venation. Plant J.

[58] Turkina MV, Kargul J, Blanco-Rivero A, Villarejo A, Barber J, Vener AV (2006). Environmentally modulated phosphoproteome of photosynthetic membranes in the green alga *Chlamydomonas reinhardtii*. Mol Cell Proteomics.

[59] Fan J, Yan C, Andre C, Shanklin J, Schwender J, Xu C (2012). Oil accumulation is controlled by carbon precursor supply for fatty acid synthesis in *Chlamydomonas reinhardtii*. Plant Cell Physiol.

[60] Fan J, Andre C, Xu C (2011). A chloroplast pathway for the de novo biosynthesis of triacylglycerol in *Chlamydomonas reinhardtii*. FEBS Lett.

[61] Miller R, Wu G, Deshpande RR, Vieler A, Gartner K, Li X, Moellering ER, Zauner S, Cornish AJ, Liu B (2010). Changes in transcript abundance in *Chlamydomonas reinhardtii* following nitrogen deprivation predict diversion of metabolism. Plant Physiol.

[62] Goncalves EC, Johnson JV, Rathinasabapathi B (2013). Conversion of membrane lipid acyl groups to triacylglycerol and formation of lipid bodies upon nitrogen starvation in biofuel green algae Chlorella UTEX29. Planta.

[63] Park JJ, Wang H, Gargouri M, Deshpande RR, Skepper JN, Holguin FO, Juergens MT, Shachar-Hill Y, Hicks LM, Gang DR (2015). The response of *Chlamydomonas reinhardtii* to nitrogen deprivation: a systems biology analysis. Plant J.

[64] Lohscheider JN, Rio Bartulos C (2016). Plastoglobules in algae: a comprehensive comparative study of the presence of major structural and functional components in complex plastids. Mar Genom.

[65] Shanmugabalaji V, Besagni C, Piller LE, Douet V, Ruf S, Bock R, Kessler F (2013). Dual targeting of a mature plastoglobulin/fibrillin fusion protein to chloroplast plastoglobules and thylakoids in transplastomic tobacco plants. Plant Mol Biol.

[66] Rider MH, Bertrand L, Vertommen D, Michels PA, Rousseau GG, Hue L (2004). 6-phosphofructo-2-kinase/fructose-2,6-bisphosphatase: head-to-head with a bifunctional enzyme that controls glycolysis. Biochem J.

[67] Reiland S, Messerli G, Baerenfaller K, Gerrits B, Endler A, Grossmann J, Gruissem W, Baginsky S (2009). Large-scale Arabidopsis phosphoproteome profiling reveals novel chloroplast kinase substrates and phosphorylation networks. Plant Physiol.

[68] Chollet R, Vidal J, OLeary MH (1996). Phosphoenolpyruvate carboxylase: a ubiquitous, highly regulated enzyme in plants. Annu Rev Plant Physiol Plant Molec Biol.

[69] Vidal J, Chollet R (1997). Regulatory phosphorylation of C-4 PEP carboxylase. Trends Plant Sci.

[70] Nimmo GA, Nimmo HG, Hamilton ID, Fewson CA, Wilkins MB (1986). Purification of the phosphorylated night form and dephosphorylated day form of phosphoenolpyruvate carboxylase from Bryophyllum fedtschenkoi. Biochem J.

[71] Taybi T, Patil S, Chollet R, Cushman JC (2000). A minimal serine/threonine protein kinase circadianly regulates phosphoenolpyruvate carboxylase activity in crassulacean acid metabolism-induced leaves of the common ice plant. Plant Physiol.

[72] Zhao ZX, Assmann SM (2011). The glycolytic enzyme, phosphoglycerate mutase, has critical roles in stomatal movement, vegetative growth, and pollen production in *Arabidopsis thaliana*. J Exp Bot.

[73] Meyuhas O (2008). Physiological roles of ribosomal protein S6: one of its kind. Int Rev Cell Mol Biol.

[74] Ferreyra MLF, Casadevall R, Luciani MD, Pezza A, Casati P (2013). New evidence for differential roles of L10 ribosomal proteins from Arabidopsis. Plant Physiol.

[75] Wool IG (1996). Extraribosomal functions of ribosomal proteins. Trends Biochem Sci.

[76] Webster C, Gaut RL, Browning KS, Ravel JM, Roberts JKM (1991). Hypoxia enhances phosphorylation of eukaryotic initiation factor-4a in maize root-tips. J Biol Chem.

[77] Gallie DR, Le H, Caldwell C, Tanguay RL, Hoang NX, Browning KS (1997). The phosphorylation state of translation initiation factors is regulated developmentally and following heat shock in wheat. J Biol Chem.

[78] Boex-Fontvieille E, Daventure M, Jossier M, Zivy M, Hodges M, Tcherkez G (2013). Photosynthetic control of Arabidopsis leaf cytoplasmic translation initiation by protein phosphorylation. PloS one.

[79] Schutz P, Bumann M, Oberholzer AE, Bieniossek C, Trachsel H, Altmann M, Baumann U (2008). Crystal structure of the yeast eIF4A-eIF4G complex: an RNA-helicase controlled by protein-protein interactions. P Natl Acad Sci USA.

[80] Das G, Shravage BV, Baehrecke EH (2012). Regulation and function of autophagy during cell survival and cell death. Cold Spring Harbor Perspect Biol.

[81] Zurita-Martinez SA, Puria R, Pan XW, Boeke JD, Cardenas ME (2007). Efficient Tor signaling requires a functional class C Vps protein complex in Saccharomyces cerevisiae. Genetics.

[82] Seaman MNJ (2012). The retromer complex—endosomal protein recycling and beyond. J Cell Sci.

[83] Turkina MV, Villarejo A, Vener AV (2004). The transit peptide of CP29 thylakoid protein in *Chlamydomonas reinhardtii* is not removed but undergoes acetylation and phosphorylation. FEBS Lett.

[84] Nordhues A, Schottler MA, Unger AK, Geimer S, Schonfelder S, Schmollinger S, Rutgers M, Finazzi G, Soppa B, Sommer F (2012). Evidence for a Role of VIPP1 in the structural organization of the photosynthetic apparatus in *Chlamydomonas*. Plant Cell.

[85] Rutgers M, Schroda M (2013). A role of VIPP1 as a dynamic structure within thylakoid centers as sites of photosystem biogenesis?. Plant Signal Behav.

[86] Huang WH, Chen QB, Zhu Y, Hu FH, Zhang LG, Ma ZX, He ZH, Huang JR (2013). Arabidopsis thylakoid formation 1 is a critical regulator for dynamics of PSII-LHCII complexes in leaf senescence and excess light. Mol Plant.

[87] Wang Z, Wang F, Hong Y, Huang J, Shi H, Zhu JK (2016). Two chloroplast proteins suppress drought resistance by affecting ROS production in guard cells. Plant Physiol.

[88] Chen YE, Yuan S, Du JB, Xu MY, Zhang ZW, Lin HH (2009). Phosphorylation of photosynthetic antenna protein CP29 and photosystem II structure changes in monocotyledonous plants under environmental stresses. Biochemistry.

[89] Xue HD, Tokutsu R, Bergner SV, Scholz M, Minagawa J, Hippler M (2015). Photosystem II subunit R is required for efficient binding of light-harvesting complex stress-related protein3 to photosystem II-light-harvesting supercomplexes in *Chlamydomonas reinhardtii*. Plant Physiol.

[90] Izumi M, Ishida H, Nakamura S, Hidema J (2017). Entire photodamaged chloroplasts are transported to the central vacuole by autophagy. Plant Cell.

[91] Huber SC, Bickett DM (1984). Evidence for control of carbon partitioning by fructose 2,6-bisphosphate in spinach leaves. Plant Physiol.

[92] Chitlaru E, Pick U (1991). Regulation of glycerol synthesis in response to osmotic changes in dunaliella. Plant Physiol.

[93] Dobrenel T, Mancera-Martinez E, Forzani C, Azzopardi M, Davanture M, Moreau M, Schepetilnikov M, Chicher J, Langella O, Zivy M (2016). The arabidopsis TOR kinase specifically regulates the expression of nuclear genes coding for plastidic ribosomal proteins and the phosphorylation of the cytosolic ribosomal protein S6. Front Plant Sci.

[94] Breitkreutz A, Choi H, Sharom JR, Boucher L, Neduva V, Larsen B, Lin ZY, Breitkreutz BJ, Stark C, Liu G (2010). A global protein kinase and phosphatase interaction network in yeast. Science.

[95] Deprost D, Yao L, Sormani R, Moreau M, Leterreux G, Nicolai M, Bedu M, Robaglia C, Meyer C (2007). The Arabidopsis TOR kinase links plant growth, yield, stress resistance and mRNA translation. EMBO Rep.

[96] Williams AJ, Werner-Fraczek J, Chang IF, Bailey-Serres J (2003). Regulated phosphorylation of 40S ribosomal protein S6 in root tips of maize. Plant Physiol.

[97] Perez-Perez ME, Couso I, Crespo JL (2017). The TOR signaling network in the model unicellular green alga *Chlamydomonas reinhardtii*. Biomolecules.

[98] Loewith R, Hall MN (2011). Target of rapamycin (TOR) in nutrient signaling and growth control. Genetics.

[99] Kamada Y, Funakoshi T, Shintani T, Nagano K, Ohsumi M, Ohsumi Y (2000). Tor-mediated induction of autophagy via an Apg1 protein kinase complex. J Cell Biol.

[100] Kawamata T, Kamada Y, Kabeya Y, Sekito T, Ohsumi Y (2008). Organization of the pre-autophagosomal structure responsible for autophagosome formation. Mol Biol Cell.

[101] Kabeya Y, Kamada Y, Baba M, Takikawa H, Sasaki M, Ohsumi Y (2005). Atg17 functions in cooperation with Atg1 and Atg13 in yeast autophagy. Mol Biol Cell.

[102] Hughes Hallett JE, Luo X, Capaldi AP (2014). State transitions in the TORC1 signaling pathway and information processing in *Saccharomyces cerevisiae*. Genetics.

[103] Yeasmin AM, Waliullah TM, Kondo A, Kaneko A, Koike N, Ushimaru T (2016). Orchestrated action of PP2A antagonizes Atg13 phosphorylation and promotes autophagy after the inactivation of TORC1. PLoS ONE.

[104] Moellering ER, Benning C (2010). RNA interference silencing of a major lipid droplet protein affects lipid droplet size in *Chlamydomonas reinhardtii*. Eukaryot Cell.

[105] Staudinger C, Mehmeti-Tershani V, Gil-Quintana E, Gonzalez EM, Hofhansl F, Bachmann G, Wienkoop S (2016). Evidence for a rhizobia-induced drought stress response strategy in *Medicago truncatula*. J Proteom.

[106] Porra RJ, Thompson WA, Kriedemann PE (1989). Determination of accurate extinction coefficients and simultaneous-equations for assaying chlorophyll-a and chlorophyll-B extracted with 4 different solvents—verification of the concentration of chlorophyll standards by atomic-absorption spectroscopy. Biochem Biophys Acta.

[107] Furuhashi T, Nukarinen E, Ota S, Weckwerth W (2014). Boron nitride as desalting material in combination with phosphopeptide enrichment in shotgun proteomics. Anal Biochem.

[108] Lohse M, Nagel A, Herter T, May P, Schroda M, Zrenner R, Tohge T, Fernie AR, Stitt M, Usadel B (2014). Mercator: a fast and simple web server for genome scale functional annotation of plant sequence data. Plant Cell Environ.

[109] Cox J, Mann M (2008). MaxQuant enables high peptide identification rates, individualized p.p.b.-range mass accuracies and proteome-wide protein quantification. Nat Biotechnol.

[110] Cox J, Neuhauser N, Michalski A, Scheltema RA, Olsen JV, Mann M (2011). Andromeda: a peptide search engine integrated into the MaxQuant environment. J Proteome Res.

[111] Olsen JV, Blagoev B, Gnad F, Macek B, Kumar C, Mortensen P, Mann M (2006). Global, in vivo, and site-specific phosphorylation dynamics in signaling networks. Cell.

[112] Sun XL, Weckwerth W (2012). COVAIN: a toolbox for uni- and multivariate statistics, time-series and correlation network analysis and inverse estimation of the differential Jacobian from metabolomics covariance data. Metab Off J Metab Soc.

[113] Saeed AI, Bhagabati NK, Braisted JC, Liang W, Sharov V, Howe EA, Li J, Thiagarajan M, White JA, Quackenbush J (2006). TM4 microarray software suite. Methods Enzymol.

[114] Saeed AI, Sharov V, White J, Li J, Liang W, Bhagabati N, Braisted J, Klapa M, Currier T, Thiagarajan M (2003). TM4: a free, open-source system for microarray data management and analysis. Biotechniques.

[115] Shannon P, Markiel A, Ozier O, Baliga NS, Wang JT, Ramage D, Amin N, Schwikowski B, Ideker T (2003). Cytoscape: a software environment for integrated models of biomolecular interaction networks. Genome Res.

