# Supplementary figures and images for: Quantitative in vivo phosphoproteomics reveals reversible signaling processes during nitrogen starvation and recovery in the biofuel model organism Chlamydomonas reinhardtii
Source: Biotechnol Biofuels. 2017 Nov 28;10:280. doi: 10.1186/s13068-017-0949-z (PMC5704542; doi:10.1186/s13068-017-0949-z)

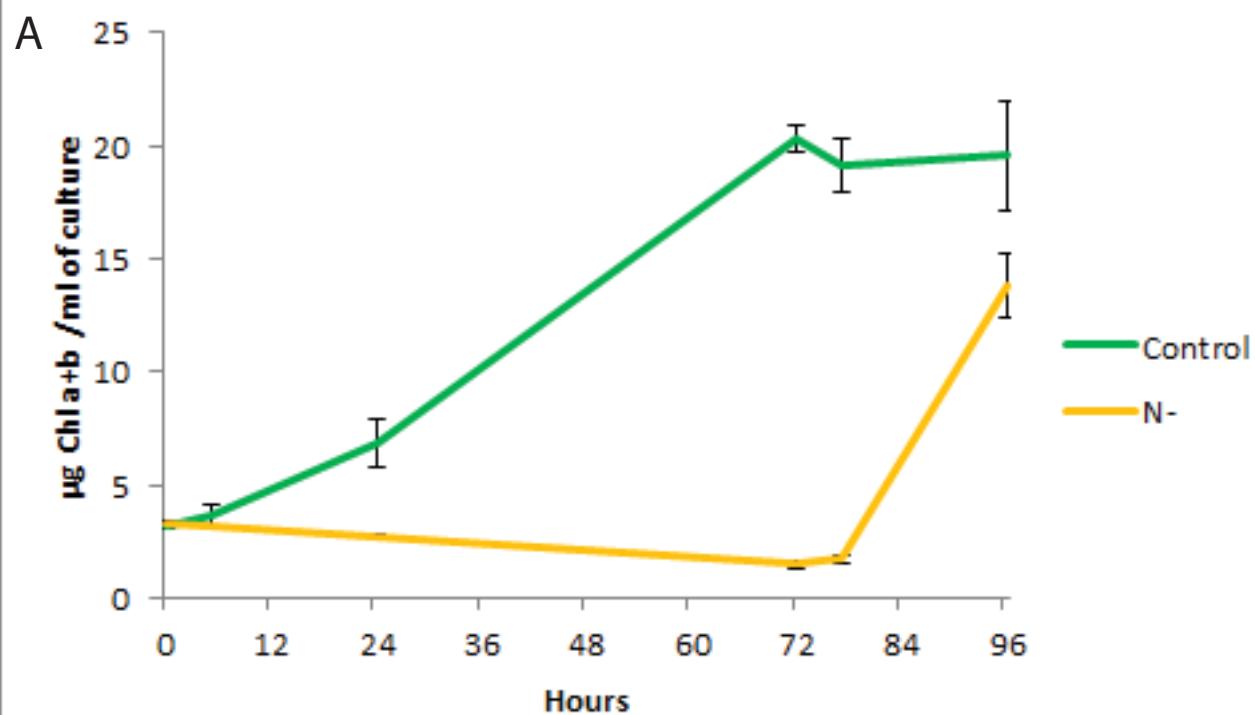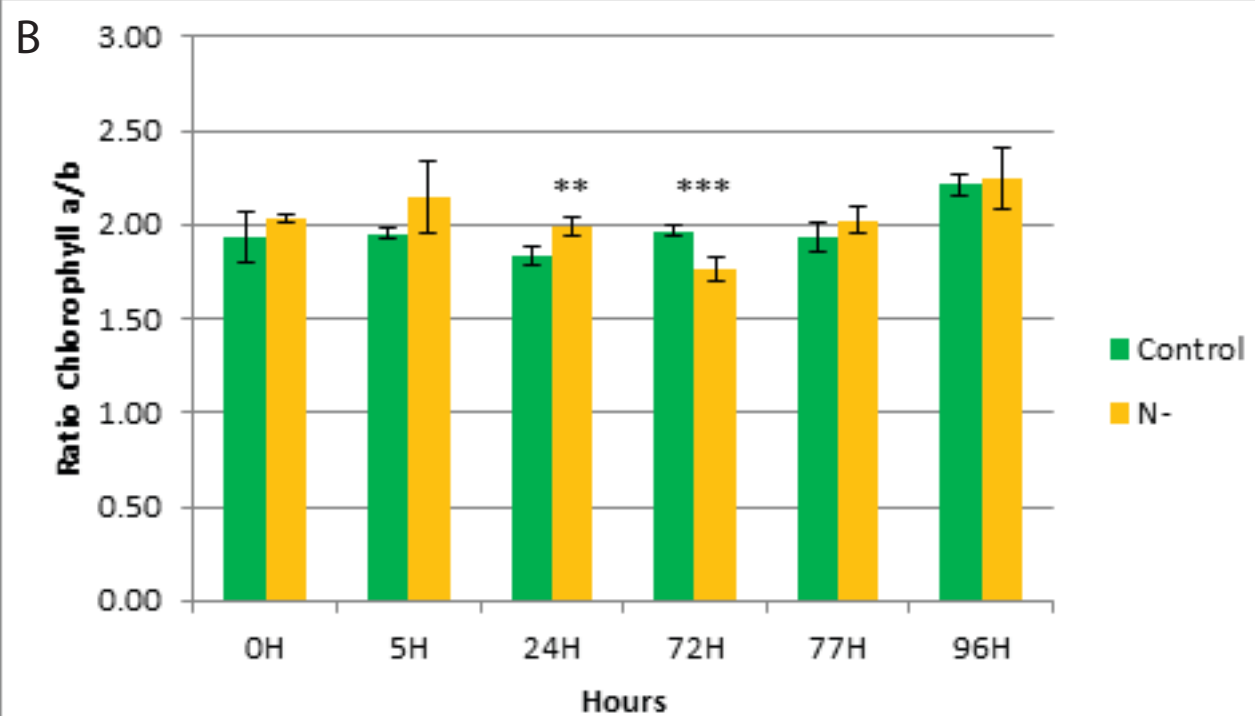

Supplement: Supplementary file 1 — Additional file 1: Figure S1. Additional data on Chlorophyll measurement. (A) Chlorophyll [a+b] concentration by milliliter during nitrogen depletion (0 h to 72 h) and N repletion (72 h to 96 h). (B) Ratio between Chlorophyll a and Chlorophyll b (p < 0,05 = *; p < 0,001 = ***). [file 13068_2017_949_MOESM1_ESM.pdf]

A

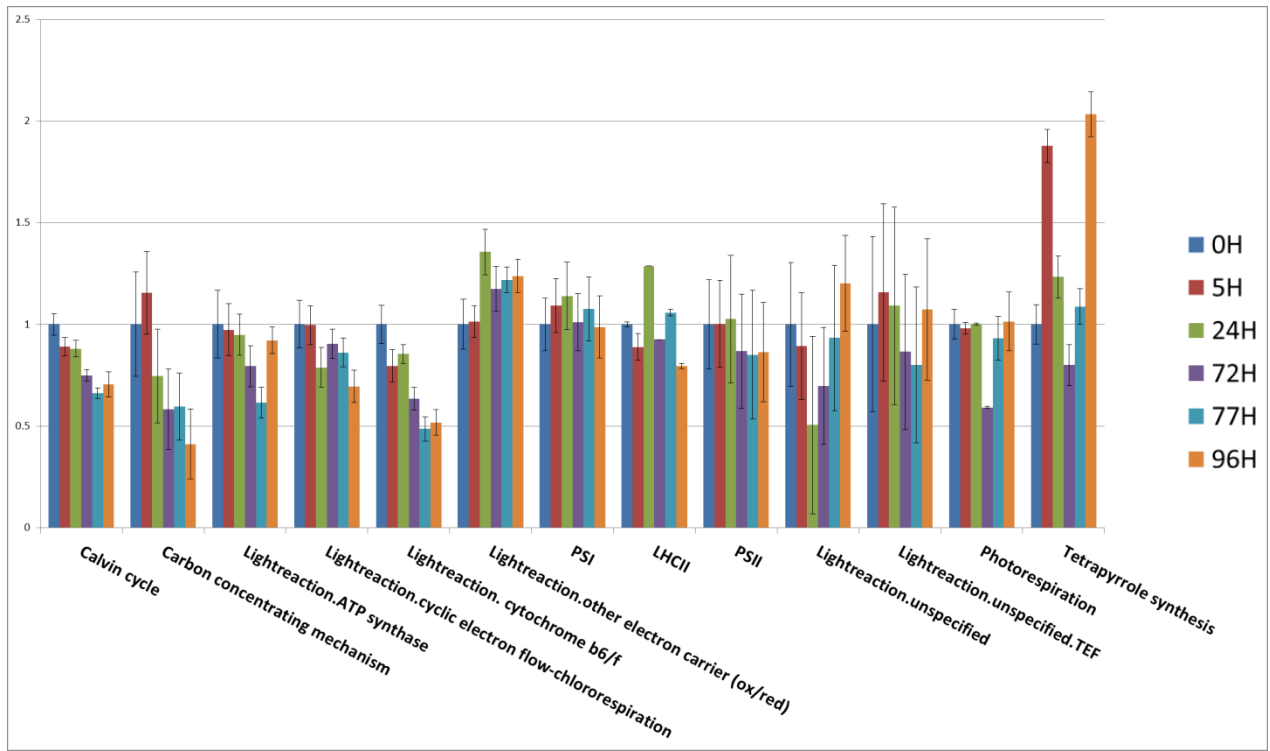

B

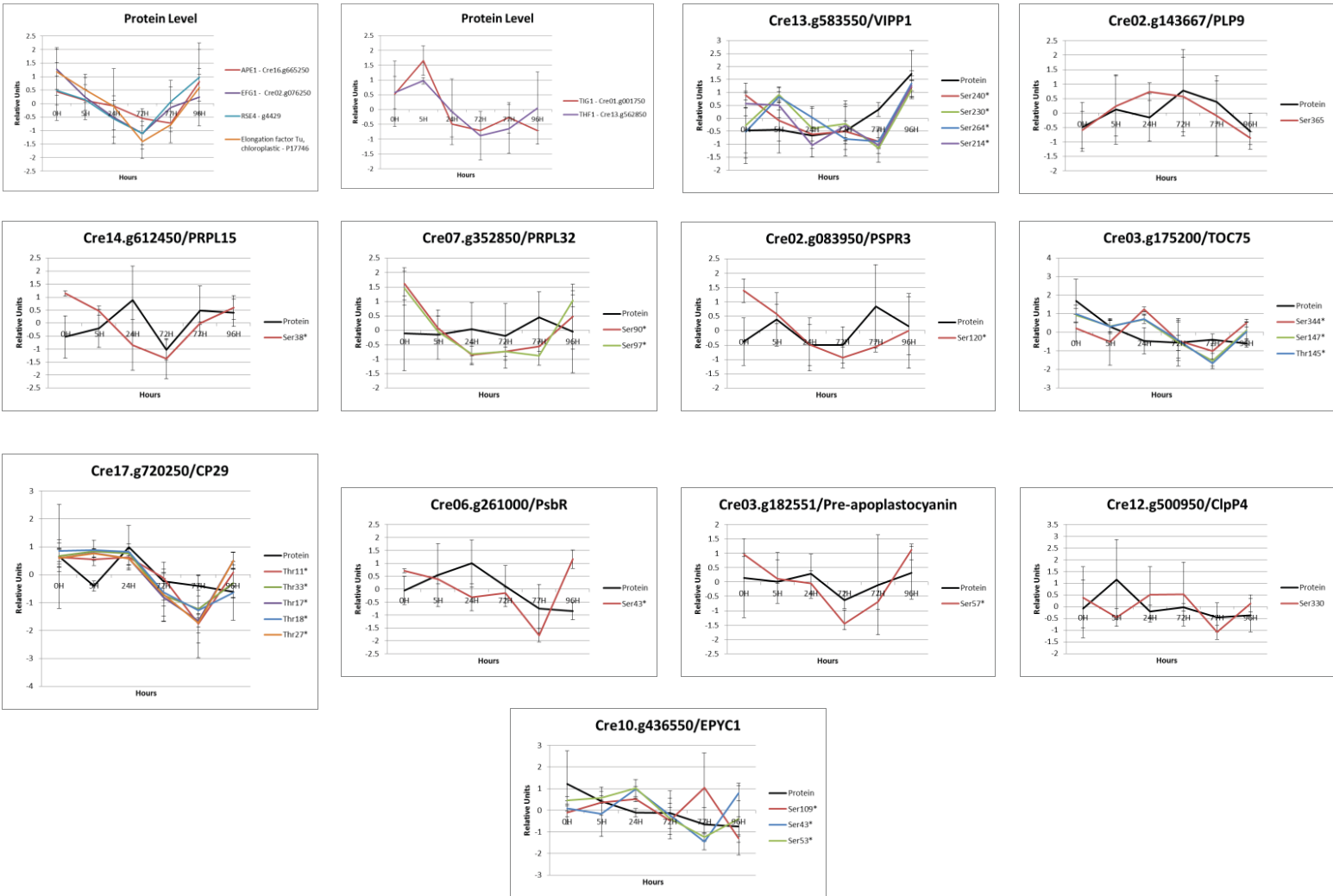

Supplement: Supplementary file 3 — Additional file 3: Figure S2. Changes in functional group as well as in phosphorylations level related to photosynthesis (A) Mean of the proteins expression level belonging to sub-functional categories of photosynthetic related protein [7]. (B) Data represents Z-transformed normalized abundances of protein and phosphopeptides belonging to photosynthesis (n = 3 for phosphopeptides and n = 4 for protein level), Phosphosites followed by * are significant (Additional file 2: Table S2). Protein levels are based on data obtained previously [7]. [file 13068_2017_949_MOESM3_ESM.pdf]

A

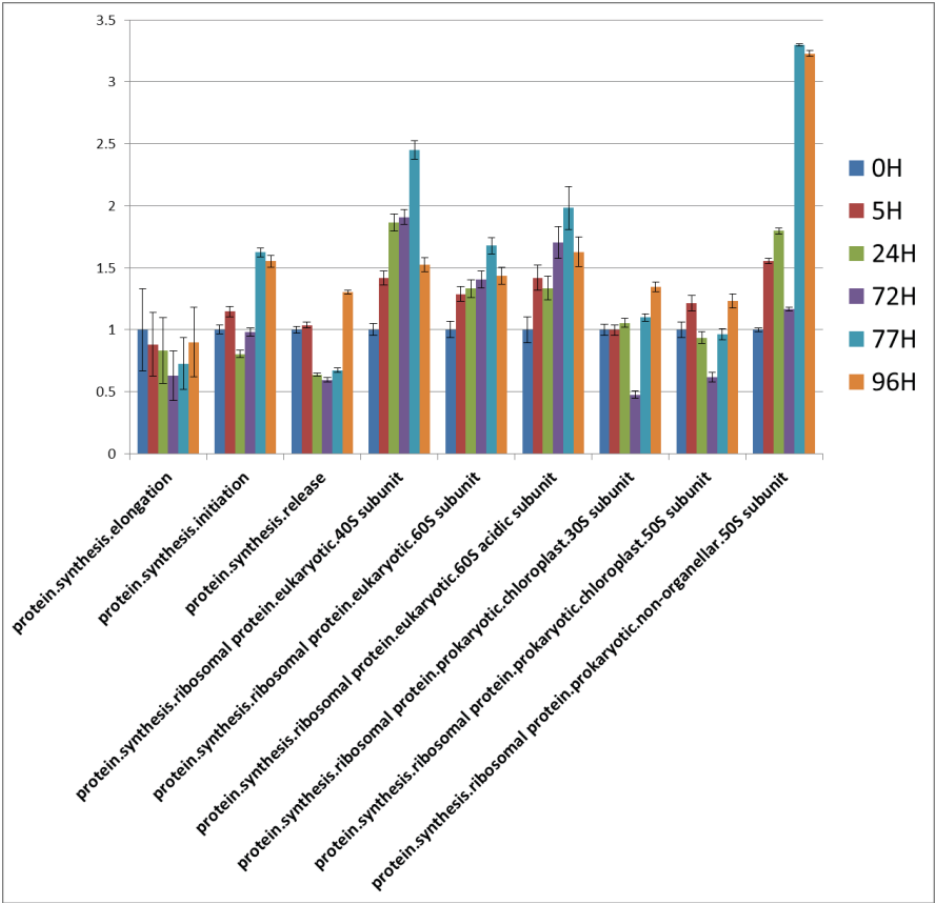

B

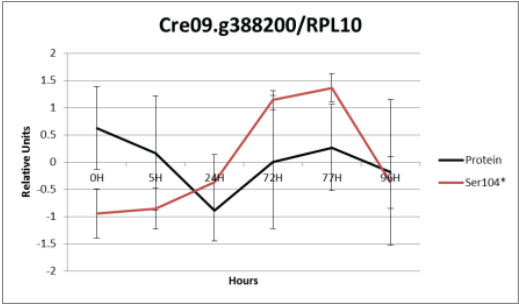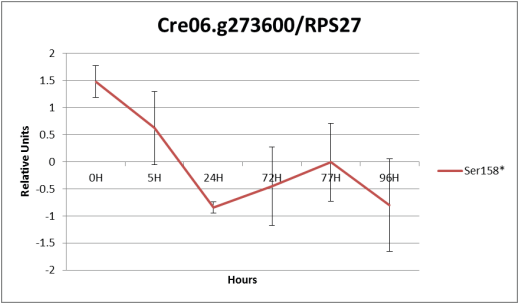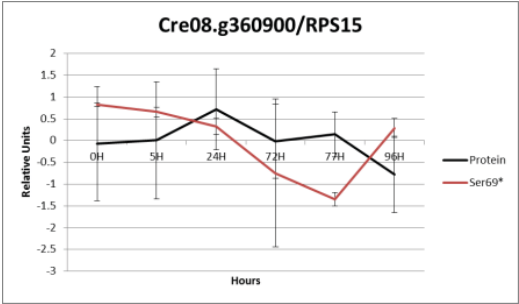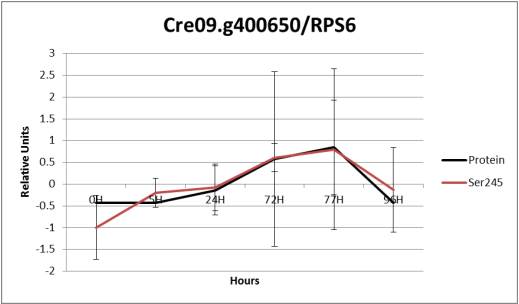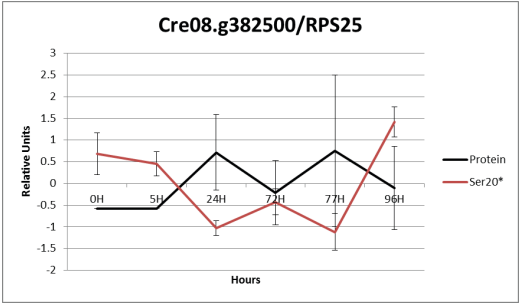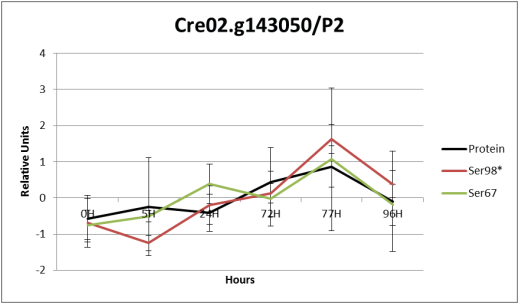

Supplement: Supplementary file 4 — Additional file 4: Figure S3. Changes in functional group as well as in phosphorylations level related to Protein synthesis (A) Mean of the proteins expression level belonging to a sub-functional category of protein synthesis related protein [7]. (B) Protein level and phosphosites level from specific protein belonging to protein synthesis category. Data represents Z-transformed normalized abundances of protein and phosphopeptides (n = 3 for phosphopeptides and n = 4 for protein level), phosphosites followed by * are significant (Additional file 2: Table S2). [file 13068_2017_949_MOESM4_ESM.pdf]

A

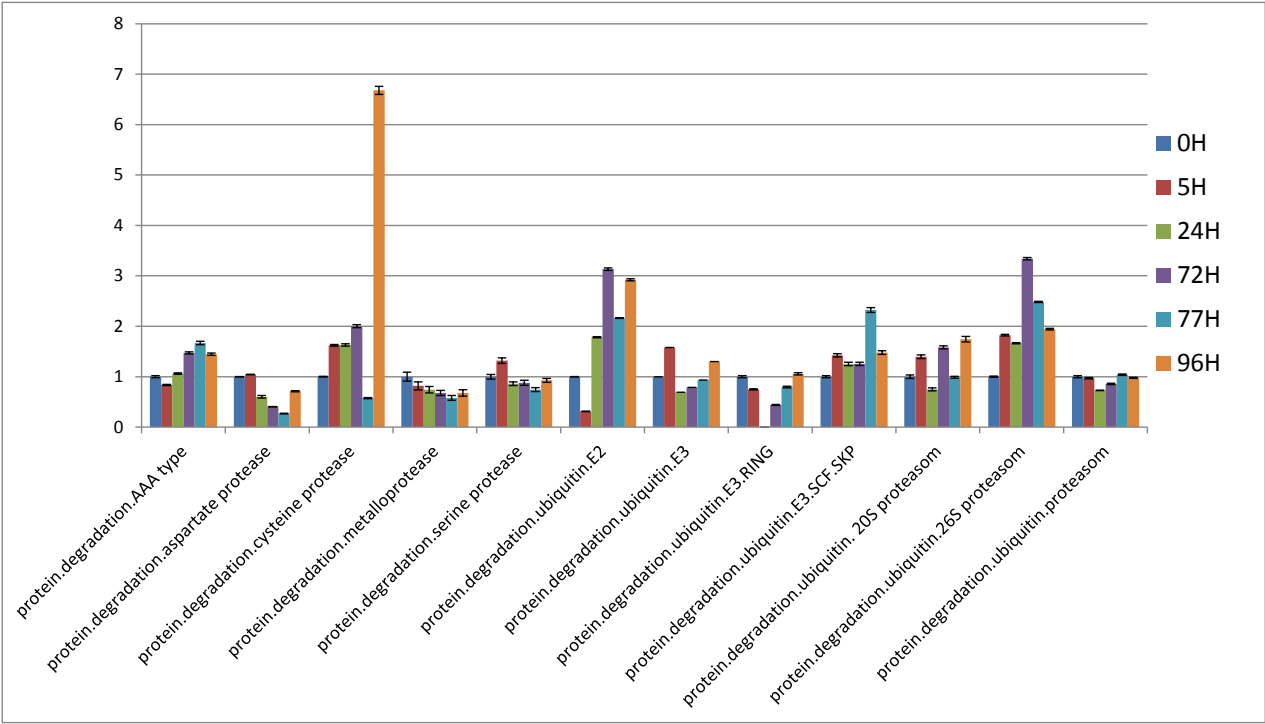

B

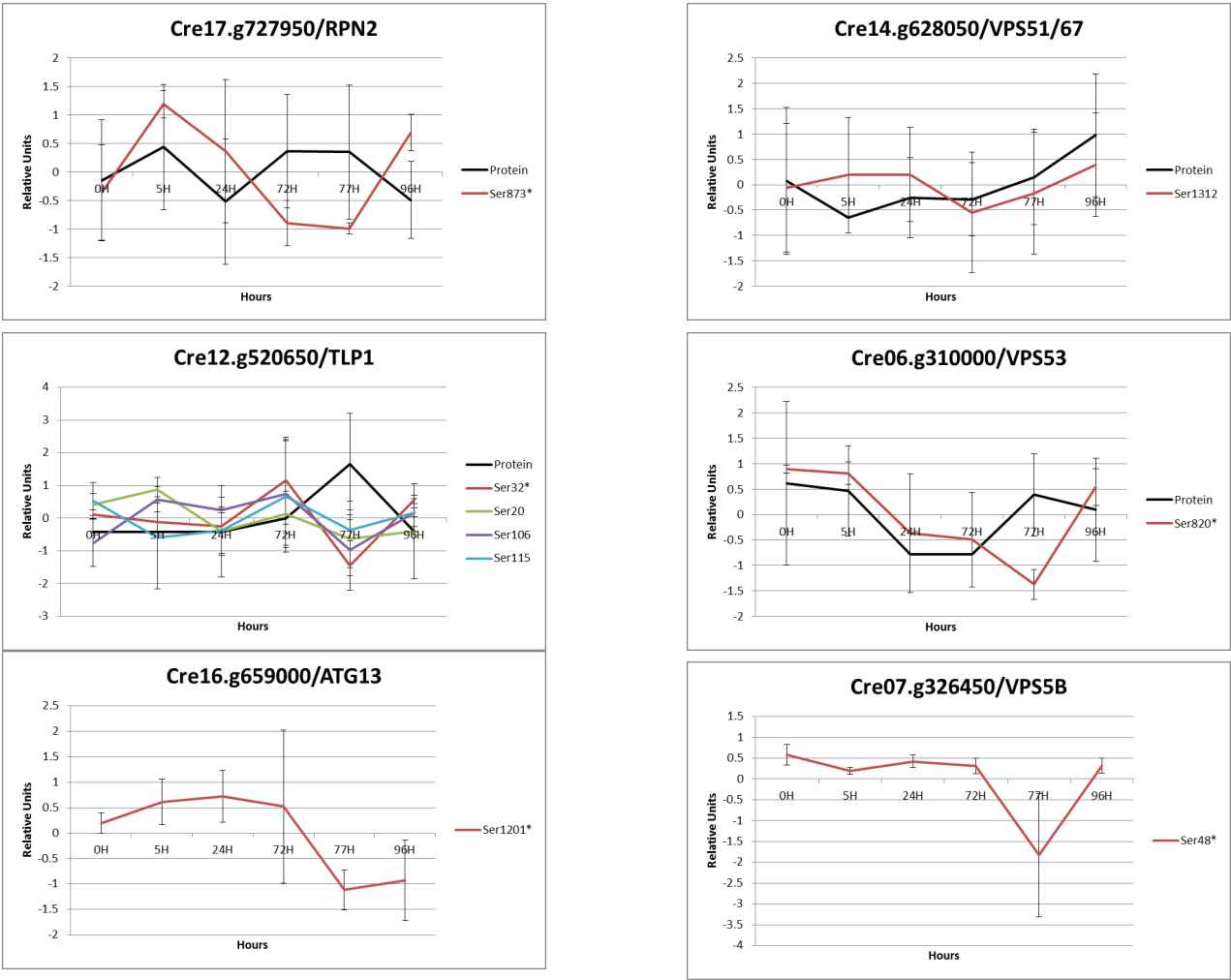

Supplement: Supplementary file 5 — Additional file 5: Figure S4. Changes in functional group as well as in phosphorylations level related to protein degradation (A) Mean of the proteins expression level belonging to a sub-functional category of protein degradation related protein [7]. (B) Protein level and phosphosites level from specific protein belonging to protein degradation category. Data represents Z-transformed normalized abundances of protein and phosphopeptides (n = 3 for phosphopeptides and n = 4 for protein level), phosphosites followed by * are significant (Additional file 2: Table S2). [file 13068_2017_949_MOESM5_ESM.pdf]

### CP29 protein level and CP29 Thr7 phosphopeptide

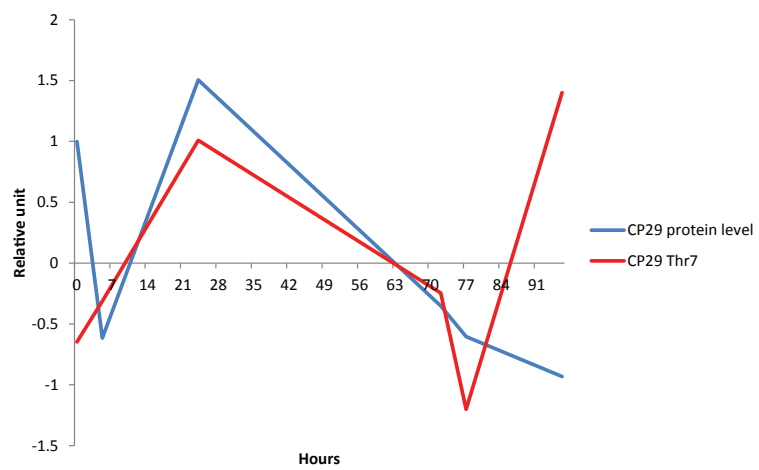

Supplement: Supplementary file 6 — Additional file 6: Figure S5. Dynamic of Threonine 7 phosphorylation level from CP29. CP29 Protein level and CP29-thr7 phosphosite level was plot together. Data represents Z-transformed normalized abundances of protein and phosphopeptides (n = 3 for phosphopeptides and n = 4 for protein level). [file 13068_2017_949_MOESM6_ESM.pdf]
